# Let’s make a mess, maybe no one will notice. The impact of bioturbation activity on the urn fill condition

**DOI:** 10.1371/journal.pone.0274068

**Published:** 2022-09-02

**Authors:** Agata Hałuszko, Marcin Kadej, Grzegorz Gmyrek, Maciej Guziński

**Affiliations:** 1 Archeolodzy.org Foundation, Wrocław, Poland; 2 Institute of Archaeology, University of Wrocław, Wrocław, Poland; 3 Department of Forensic Biology and Entomology, Faculty of Biological Sciences, University of Wrocław, Wrocław, Poland; 4 Usługi Archeologiczne Grzegorz Gmyrek, Kalisz, Poland; 5 Department of General and Interventional Radiology and Neuroradiology, Wrocław Medical University, Wrocław, Poland; New York State Museum, UNITED STATES

## Abstract

The research was carried out at the cremation cemetery of the Lusatian culture in Wtórek, Ostrów Wielkopolski district, Wielkopolska province, Poland. Contrary to the so-far-studied topics related to the CT imaging of burnt bones and their virtual exploration, we concentrated on the analysis of the structures formed by the soil fauna activity in the fills of urns and additional vessels, and reconstruction of the dynamics of the ecosystem variability within the cemetery area based on thereof. We also demonstrated the impact of macrofaunal activity on stratigraphy and bone fragmentation. From the total of 222 excavated burials in 18 urns and one additional vessel, the remains of macrofauna or its bioturbation activity were identified. Out of 19 vessels subjected to CT examinations, traces of macrofaunal activity were demonstrated in 13: in five vessels animal bioturbative activity was not observed and in one, observations was impossible (due to significant metal-related artifacts). In two vessels both macrofaunal remains and traces of activity were identified. Discovered bioturbations were associated with specific species or genera. Nests or their parts of the genus *Geotrupes* sp. beetles were the most frequently observed traces of macrofaunal activity. Tunnels and aestivation chambers of earthworms and chambers of the genus *Harpalus* sp. beetles filled with *Setaria* sp. caryopses were discovered. The chitinous parts of other insects and the humerus bones of the vole of the genus *Microtus* sp. were also identified. It was shown, especially due to the non-destructive method, that rodents activity had the most destructive effect on the bone stratigraphy as well as on the movement and fragmentation of the burnt bones. The chances of visualizing bioturbations decreased with time since their creation. The process of disappearance of traces of macrofaunal activity concerned both traces of rodent activity and nests set up by *Geotrupes* sp. and other species.

## Introduction

A prehistoric cinerary urn is a kind of unique natural environment (= habitat of species), subject to various influences and modifications that take place from the moment of depositing burnt remains in a vessel and burying it, to the time of unearthing the urn by archaeologists [1–3]. The uniqueness of a prehistoric urn lies in its clear separation with ceramic walls from the soil environment in which it is buried. This means that all soil processes and their effects remain "locked" inside the urn just like in a time capsule. Thanks to this, they can easily be transferred to a laboratory and subject to research and interpretation. Detailed analysis of the urn fill structure can provide information on environmental changes, especially ecological succession or ecosystem variability, which is mainly the outcome of anthropopressure. Often identification of bioturbation activity of soil fauna can have an enormous impact on the assessment of anthropological parameters, especially pseudopathological skeletal alterations [4–6], the degree of fragmentation of bone remains [7–10] and the proper identification of the anatomical order in the urn [11–13].

Soil and bioturbation processes could remain intangible during urn exploration. This is especially true in the case of sandy fills, which, on the one hand, are uniform in colour even after mixing of the layers, and, on the other hand, these fills are very loose and thus make the layers difficult to see. Use of computed tomography and the analysis of individual CT slices as well as 3D reconstruction address these problems. Some of the soil processes, such as soil pressure and its effect on the stratigraphy (collapse) of burnt bone remains are quite easily and unambiguously identifiable [7, 14]. An additional advantage of CT imaging is the ability to capture the proper (plastic) layers of the fill, which should be followed during mechanical exploration [14–16].

The bioturbation activity of macrofauna and mesofauna inside the urn also usually leaves clear traces visible in tomograms. However, some of the bioturbants leave surprising traces of their activity and may cause misinterpretation of changes observed on bones as well as of the bioarchaeological finds themselves [6, 17]. This study is mainly devoted to the analysis of intangible structures that can only be perceived virtually. The grounds for their existence are the remains of macrofauna found among the human bones that were obtained from the urns discovered at the cemetery in Wtórek, Ostrów Wielkopolski district, Poland. Some of the animals discovered there could serve as a reason for misleading interpretation that could have even been transposed into the religious and symbolic sphere. The main research problem undertaken in this work is the analysis of the observed structures remaining after the faunal activity in the urn fills and, based on them, the reconstruction of the dynamics of ecosystem variability taking place within the cemetery as the outcome of anthropopressure impact. Last, but not least, this work also aims to demonstrate the significant impact of the macrofaunal activity on the stratigraphy and the degree of burnt bones fragmentation.

### Archaeological background and studies

The Lusatian culture belongs to the Urnfield cultures milieu and territorially it encompasses mainly the areas of today’s Poland, eastern Germany, the Czech Republic, Slovakia and western Ukraine. Within the area of Poland, it was present from the Late Bronze Age to the Early Iron Age, viz. around 1300–400 BC [18, 19]. It is considered that as an archaeological culture it was formed as the result of the transformation of local communities under the influence of a strong ideological and political movement manifested by the unification of the funeral rite and the widespread use of cremation as the binding method of burial [20, 21]. In the Early Iron Age, these cultural formations underwent another stage of socio-cultural transformation under the influence of the Hallstatt culture [22–26]. Therefore, it is considered that the Lusatian culture is heterogeneous and it is divided into many local groups that differ both in terms of settlement patterns and various details of the funeral rite [27]. It is often referred to as the so-called Lusatian culture, or Lusatian Urnfield culture to emphasize this heterogeneity.

Cremation cemeteries of the Lusatian culture are one of the most numerous archaeological sites research within the area of today’s Poland [23, 28]. To date, thousands of graves have been examined, from which a huge number of archaeological artefacts was obtained, including luxurious artefacts such as gold and amber items as well as iron and bronze swords [24, 29]. Many of these artefacts were subject to specialist analyses, thanks to which both the technology of their production and their provenance were identified [30, 31]. The research on prehistoric cinerary urns focuses largely on the anthropological and zooarchaeological analysis of the bone remains preserved in them and accompanied by grave goods consisting of various types of metal, glass, ceramic artefacts and other items.

The cemetery in Wtórek (51.639414° N, 17.875440°E) was discovered by accident in 1999 by then the owner of a farm located near the cemetery [32]. Archaeological surface survey verification preceding the excavations confirmed the existence in this place of a necropolis of the Lusatian culture. Before the excavations, there was an arable field at the site of the cemetery. From the oral information obtained from the owner of the field, it was learned that in the 1950s there had been a meadow at this spot, on which horses and cows had been fed.

Wide-scale archaeological excavations were of commercial nature and were carried out between 2012 and 2016 by a consortium of two companies: Usługi Archeologiczne Grzegorz Gmyrek and MBL Leszek Ziąbka. The works were commissioned by GDDKiA (Eng. the General Directorate for National Roads and Motorways). They were carried out in connection with the construction of the Ostrów Wielkopolski bypass and the reconstruction of the district road from Ostrów Wielkopolski to Grabów nad Prosną. All necessary permits were obtained for the described study, which complied with all relevant regulations. Excavation permits were given by the Wojewódzki Urząd Ochrony Zabytków w Poznaniu, Delegatura w Kaliszu (Eng. Voivodeship Monuments Protection Office in Poznań, branch office in Kalisz), permit nos. 95/2012/C, 78/2013/C, 43/2014/C, 137/2015/C, 235/2015/C, 106/2016/C. The permits also included research related to the analyses of archaeological and bioarchaeological materials.

**Fig 1:**
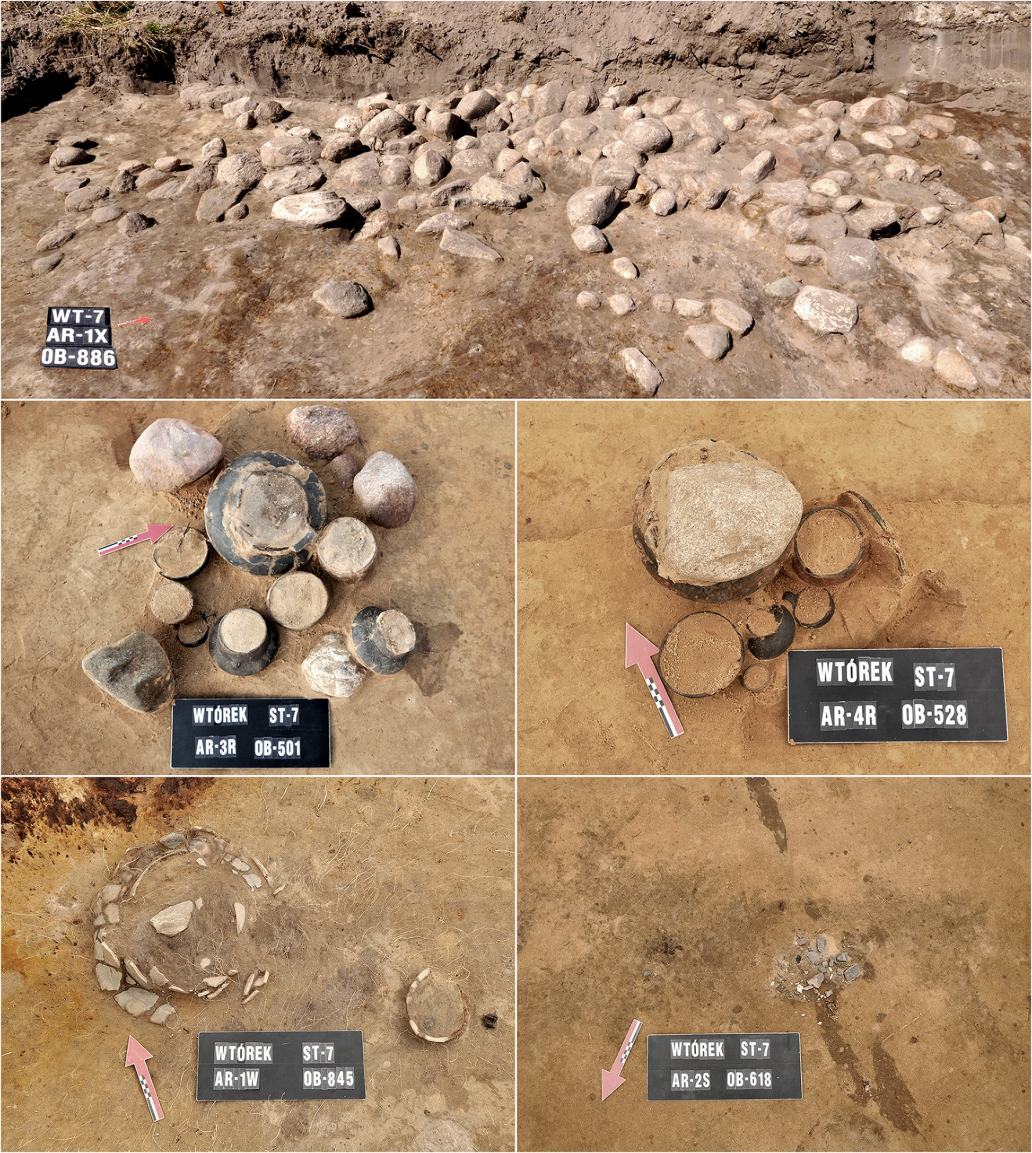
Burials *in situ* at the cemetery in Wtórek, Greater Poland, Poland. At the top, stone pavement covering the grave 886. In the middle row—well-preserved graves 501 and 528, and at the bottom—poorly preserved burial 845 and very badly preserved burial 618.

As the outcome of the excavations carried out on over 220 ares, the presence of a settlement and a cemetery of the Lusatian culture was recorded, as well as traces of medieval and modern settlement. At the necropolis of our interest here, 222 cremation graves of varying degrees of preservation were discovered within the area of approximately 27 ares. Most of the burials were destroyed due to the shallow depth of deposition and resulting from it damage caused by agricultural activities (Fig 1).

**Fig 2:**
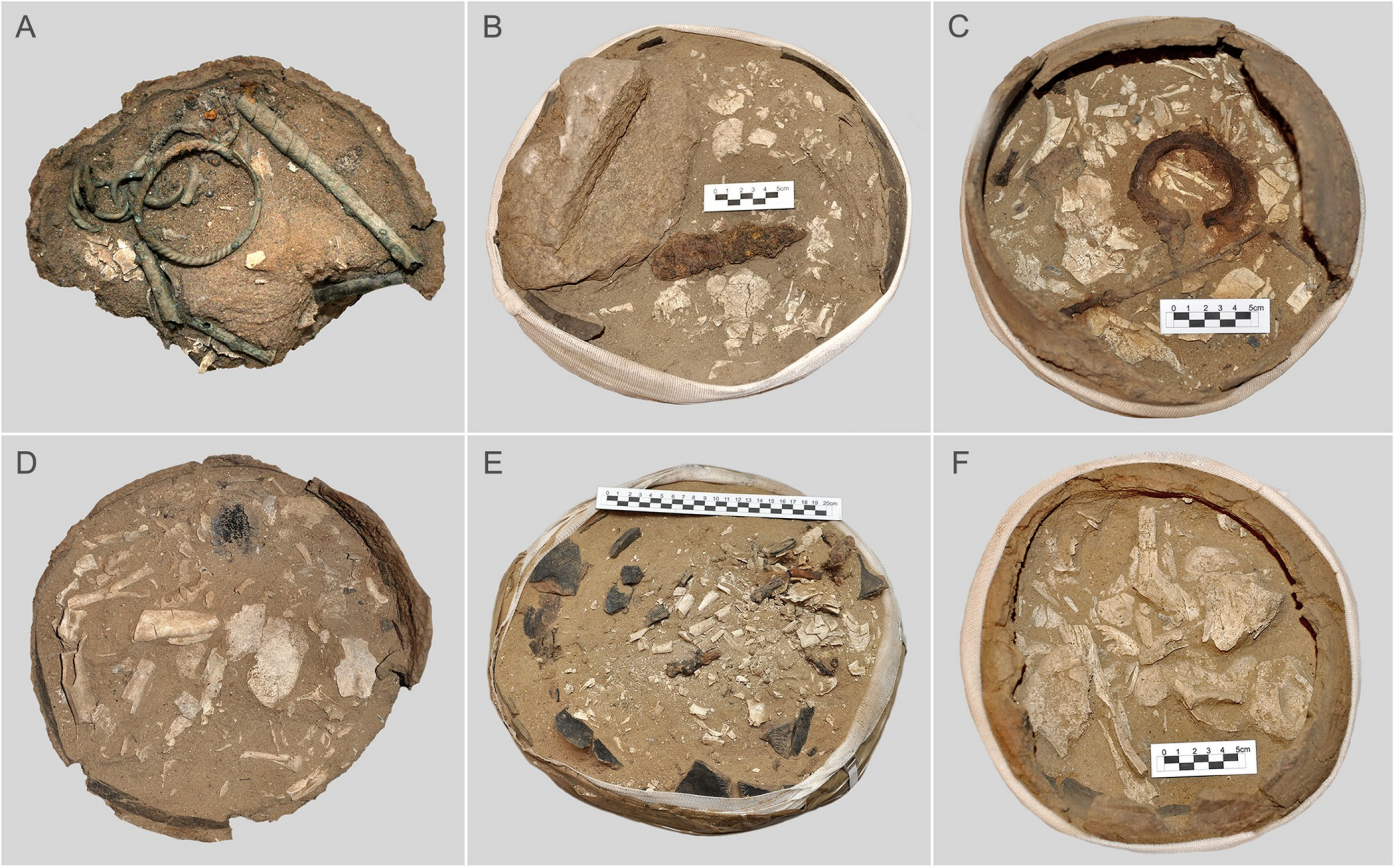
Various forms of depositing burnt human remains and grave goods consisting of metal artefacts. Metal items covering the burnt bones in the urn from burial 506 (A), 544 (B), 578 (C), the bones of the skull vault located in the top layer of the urn from grave 537 (D), and fragments of the shafts and epiphyses of long bones located in the upper-most layers of the urns from graves 858 (E) and 722 (F).

Only the so-called flat graves, currently with no perceptible surface features were recorded. In a few cases, the outlines of burial pits were preserved (16.7%). Most of the discovered burials were urn graves (82.9%), in which human bones had been deposited in a vessel—cinerary urn. In the case of the remaining burials, probably an organic container, the remains of which did not survive, had been used as an urn. Some of the graves had various types of stone structures (27.1%), which were manifested in the form of stone pavements covering burials, burial chambers and stone lining, as well as individual large stones located within the grave pit (Fig 1). Grave inventory usually consisted of ceramic vessels, whose number varied from 1 to 15. In the case of approx. 19.4% of the burials, no additional vessels (secondary vessels) were present apart from the urn. In addition to the ceramic grave goods, the presence of items made of bronze, iron, glass, stone and animal bone was confirmed (Fig 2). Thanks to the presence of metal items, particularly ferrous ones, several pieces of textile fabric were secured in the form of a textile pseudomorph, which had been formed by mineralization of the original fibres [33, 34]. The presence of such forms was found in about 7% of the burials. Textile pseudomorphs were separately examined and subject to a scanning electron microscope (SEM) imaging to identify the type of weave and the raw material of the fabrics used in the funeral ceremony. In this study, the results of the textile pseudomorph analyses were limited to the most important information necessary for further investigation.

On the basis of the discovered archaeological artefacts, such as ceramic vessels and/or metal objects, the chronology of the features was established in accordance with the system developed by J. Kostrzewski and adopted for Polish lands [35]. The oldest burials were dated to the Late Bronze Age (the IV and V period of the Bronze Age–IV BA and V BA), while the youngest—to the Early Iron Age (Hallstatt period C–Ha C), which is within the range of circa 1200–400 BC [36]. It should be emphasized that only a part of the burial ground was subjected to archaeological investigation and perhaps other unexplored zones would extend this chronology.

### Analyses of burnt osteological remains

Analyses of burnt osteological remains is usually very difficult due to the fragility of the bones and a significant degree of their fragmentation [8, 37–39]. Interestingly, recently bioarchaeological analyses of burnt osteological remains became the subject of many scientific papers. Some of them focus on the research and development of methods for assessing sex and age at death on the basis of burnt human bones [40–44]. The accuracy of picking human remains from the funeral pyre is also studied, based on the analysis of the weight of human bones and various bone fractions, such as skull bones or long bones [45–47]. Another important aspect of the research directly related to bone fragments is the analysis of bone cracking, which, by some scholars, is identified as a differentiation of the corpse treatment before burning [48, 49]. The most recent research results are dominated by strontium isotope analyses, which lead to the verification of the autochthonous/allochthonous origin of particular individuals discovered at a given cemetery [50–54]. Another intensively researched issue reflects the growing interest in non-invasive studies and visualization of entire fills of preserved urns [7, 14–16, 55–57].

More and more often, mechanical exploration of fills of vessels coming from cremation graves is preceded by non-destructive and non-invasive examinations such as computed tomography (CT) imaging and virtual 3D exploration [14–16, 55, 58–60]. Thanks to multislices CT imaging, it is possible to anatomically identify some bone fragments, determine the degree of their fragmentation, and make multiplanar measurements. In addition, it is possible to precisely locate most of the archaeological artefacts deposited in the urn. Most often, these include bronze and iron tools and ornaments, as well as additional vessels. Examination of their position in relation to the bone material, preliminary assessment of the type of metal, and determination of whether they are burnt or not present no difficulties [16, 60]. Undoubtedly, conducting non-invasive research before the mechanical exploration of an urn increases the precision of anthropological analyses [14, 16]. All these elements enhance our knowledge about funeral practices, and, by improving the quality of anthropological analyses, they also increase the chances of determining the age and sex structure as well as the biological condition of the population under investigation.

**Fig 3:**
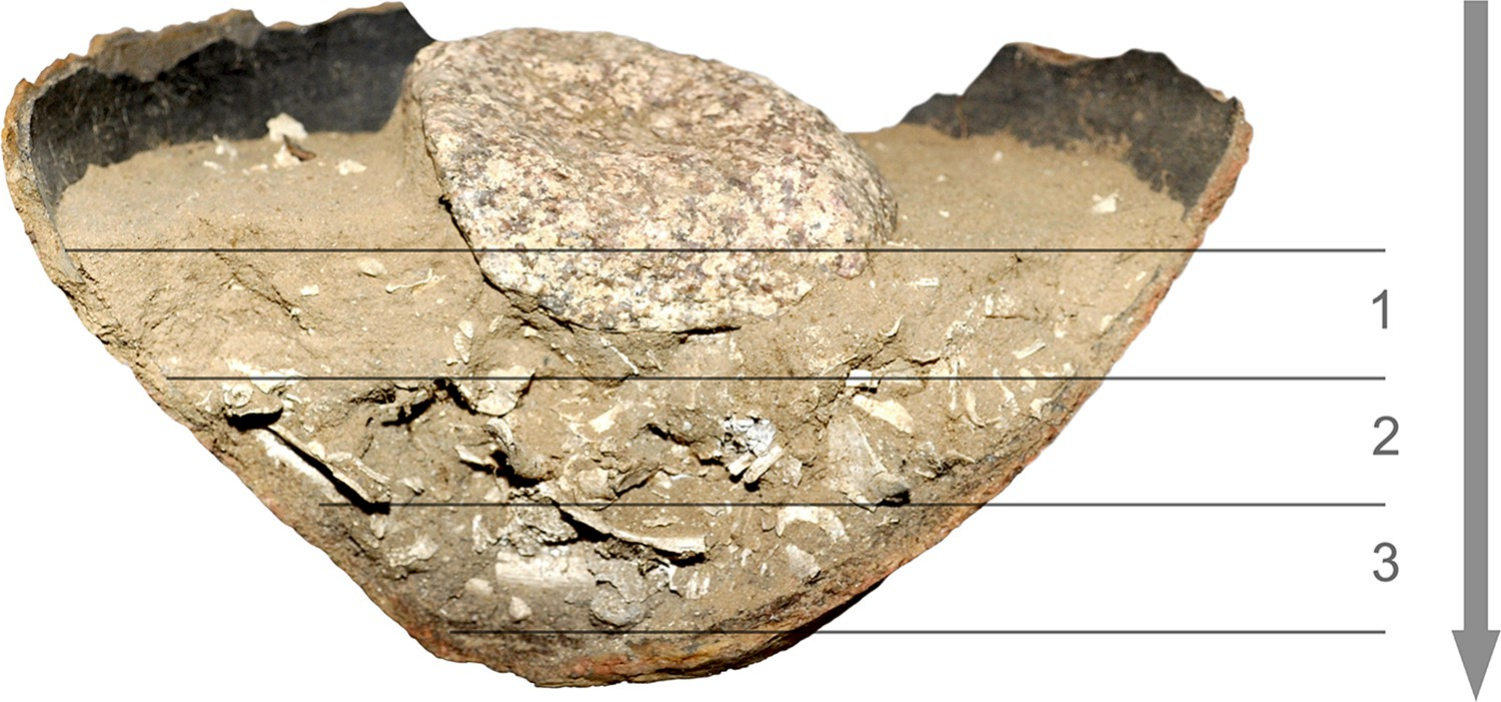
Diagram of the direction of mechanical exploration and the order of nomenclature of individual layers of the urn fills.

The exploration of the vessel fills from the Wtórek cemetery was preceded by marking the color of the soil. For this purpose the Munsell Soil Color Chart was used. For all studied vessel fills, the color notation was uniform and marked as light brown (7.5YR 6/4) and light yellow brown (10YR 6/4). Medium to coarse sand has been identified as soil filling the vessels. Urns and additional vessels (grave goods) were explored mechanically. The fills of the vessels were explored in spit layers approx. 5 cm thick, the number of which was adjusted depending on the vessel height (Fig 3). To reduce bone damage and additional bone fragmentation the exploration was performed using soft brushes and wooden tools. Some of the vessels were crushed, and hence their fills were not divided into layers. The spit layer exploration was primarily aimed at determining the exact location of archaeological artefacts in relation to the burnt bones, collecting information about the stratigraphy of bone arrangement and its disturbance by bioturbative factors, and determining the existence or absence of the so-called anatomical order of the human remains in urns [11–13]. In the case of burnt human remains, the anatomical order is defined as the presence of skull bones in the upper layers of the fill, i.e. near the rim of the vessel (Fig 2D), and the bones of the lower limbs in the lower layers, i.e. close to the bottom of the vessel. The individual layers were photographed and the bones collected from them were packed separately.

In the course of anthropological analyses of osteological materials from Wtórek cemetery, the presence of 257 individuals in 222 discovered graves was confirmed. Anthropological studies were conducted to estimate sex and age at death of individuals. The sex of the individuals was estimated according to the methods based on morphological dimorphism of the skull and postcranial skeleton [38, 61]. The lateral angle method was also used as a complementary method [62–66]. The age at death of non-adult individuals was determined on the dentition [67, 68] and the skeleton stage of development [68–70], while the age of adult individuals was determined on the degree of obliteration of the cranial sutures [61, 71, 72] and tooth cementum annulation [73–75]. Based on these methods the sex of most individuals was indeterminable (67,8%). Only 23,3% were identified as female and 8,9% as male. Predomination of individuals classified within the youngest age categories (30,7%) was observable.

The weight of the burnt bone remains ranged from 0.02 g to 4534.80 g. On average, the presence of 175.40 ± 315.21 g of bone was recorded per urn. A high degree of calcination was observed in bones and most of them were light yellow, white, and chalky in colour. In many cases, fragments of the cranial vault bones and individual fragments of the shafts of long bones were characterized by a different colour, most often graphite, black or by the so-called "sandwich effect" [76]. For most osteological material, it was possible to do a macroscopic evaluation of bone fractures. The types of bone warping and splitting were determined and described in accordance to D.H. Ubelaker [48] that is as: transverse fracture lines, irregular lengthwise splitting, thumbnail, and cracking and longitudinal splitting. The vast majority of cracks was of a transverse and irregular type (90.7%). Only in 3 cases the presence of longitudinal splitting was noted.

**Fig 4:**
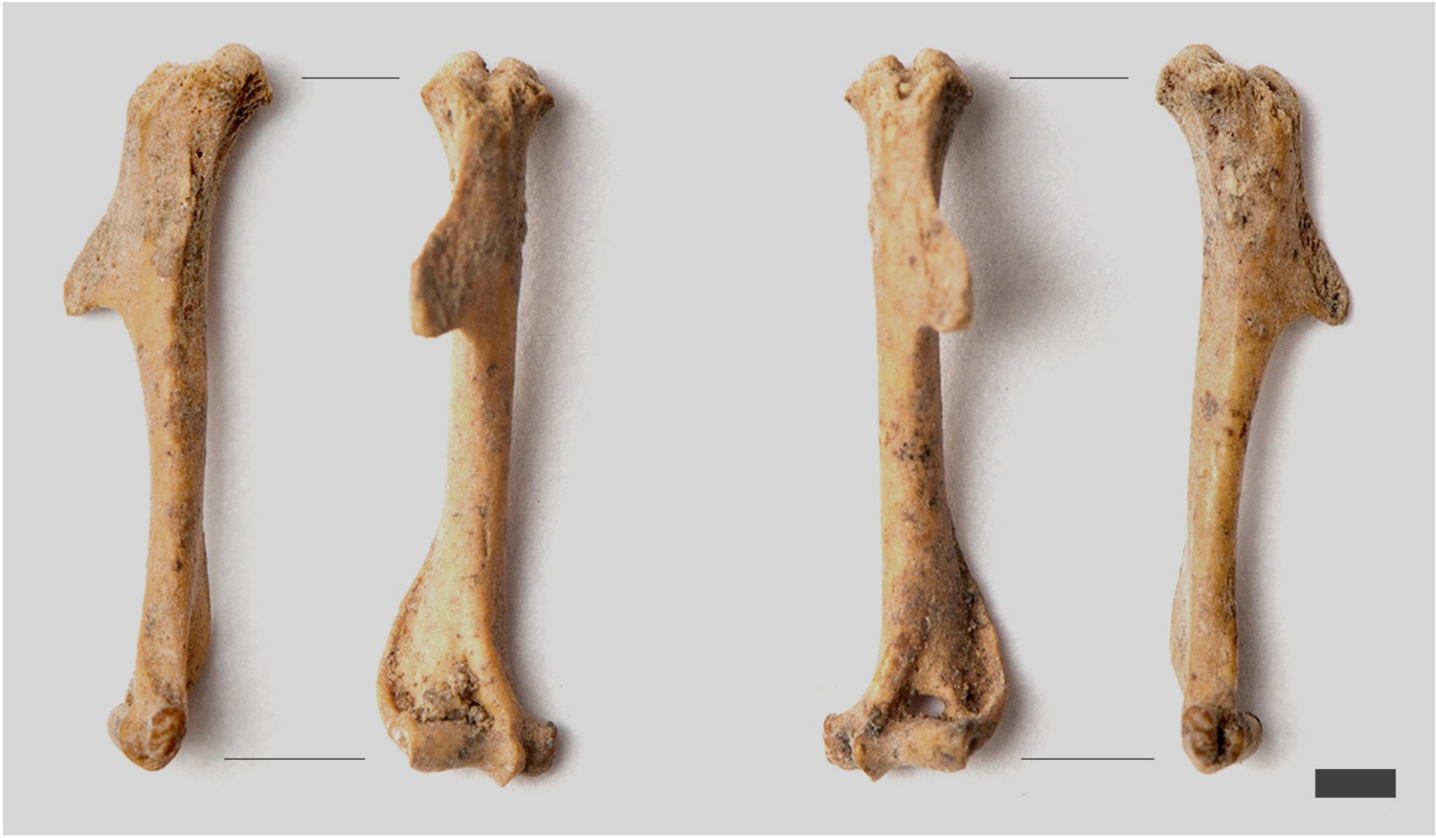
Identified remains of a rodent of the genus *Microtus* sp. in the urn from burial 853. The right and left humerus of the vole in the anterior and medial plane.

Throughout the course of anthropological analyses, animal bones were separated and subjected to independent zooarchaeological analyses. In general, animal bones were present in about 9% of the burials and most of them were burnt animal bones of domestic mammals. Only in the urn from burial 853, dated to the V BA, in which an adult of unspecified sex was discovered, two unburnt humeri (right and left) of a modern vole were found (Fig 4).

**Fig 5:**
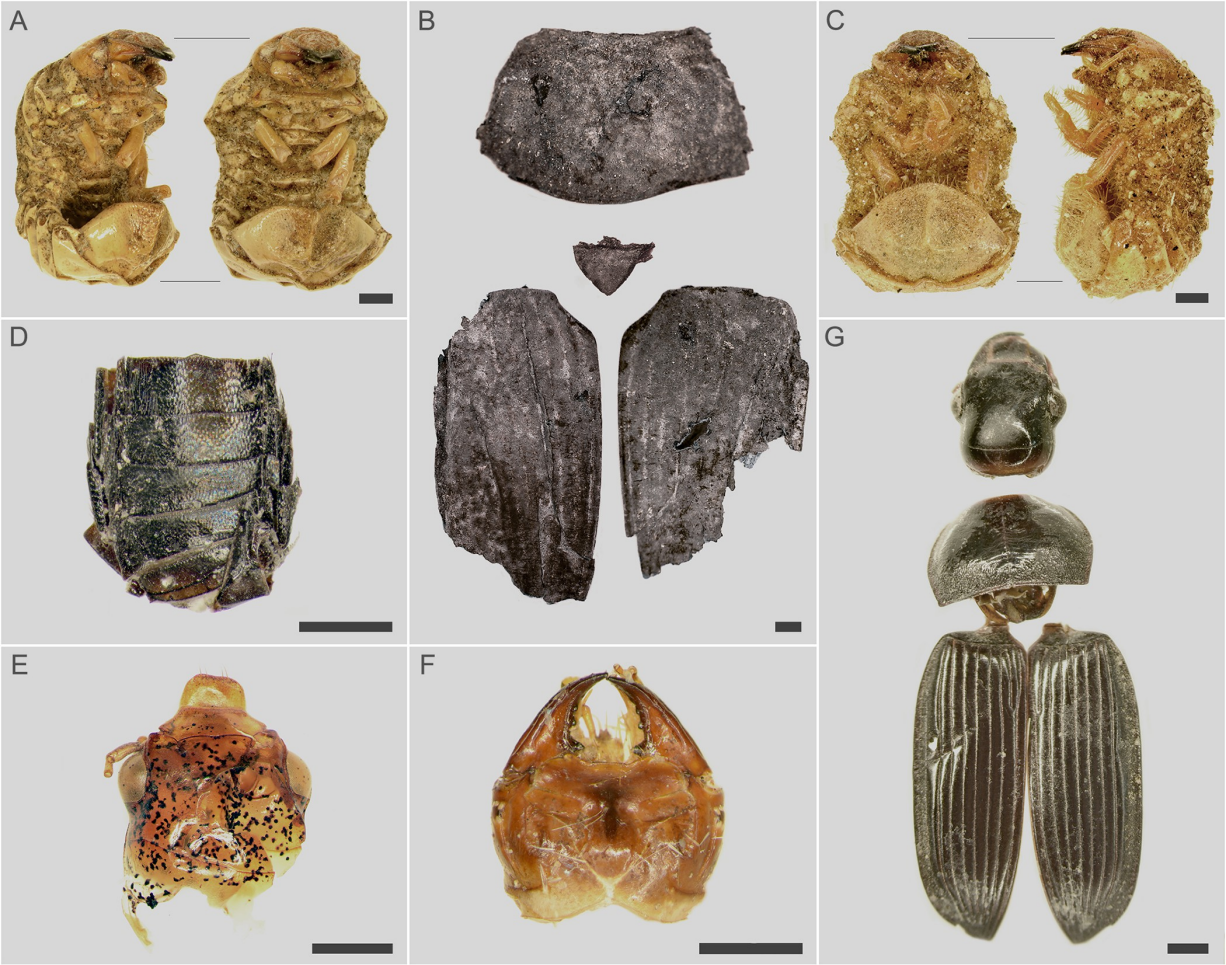
Insect remains identified in the urns. Desiccated larva from the urn from burial 504 (A), fragments of *Geotrupes stercorarius* from the urn from burial 506 (B), desiccated larva from the urn from burial 507 (C), a fragment of an adult beetle from the family *Staphylinidae* from the urn from burial 533 (D), head of an adult beetle *Harpalus flavescens* from the urn from burial 542 (E), head of a beetle larva of the genus *Harpalus* sp. from the urn from burial 599 (F), remains of an adult beetle *Anisodactylus nemorivagus* from the urn from the burial 615 (G).

During the conducted osteological analyses, the presence of two very well-preserved, dried-up grub-type larvae and several fragments of other insects were observed (Fig 5). The insect remains were discovered at various depth levels of the urn fills, i.e. within different layers. These materials were collected and protected against mechanical damage by inserting them into Eppendorf test tubes.

During the exploration of the urn from grave 599, two clusters of bristle grass caryopses (*Setaria* sp.) were discovered. One of them had the form of a vertical tubular corridor filled with grains.

Apart from the performed osteological analyses and mechanical exploration of the urns, non-destructive studies were carried out. For this purpose, the best-preserved pottery vessels were selected and subjected to CT scanning. The procedure and results of the non-invasive analyses are described later in the paper.

## Material and methods

From the total of 222 excavated burials, the main subjects of the research were the discovered remains of macrofauna in eight urns and their activity traces discovered in 13 from 19 vessels, which had been secured, together with their intact fills, during the excavations. The vessel fills consisted mainly of fragmented burnt osteological remains and sandy soil. In general, the constituents of the vessels fills were loosely packed and thus easily susceptible to internal structure damage, e.g. due to vibrations. Therefore, having in mind the concerns related to the destructive impact of long-distance transport causing disturbance of the primary stratigraphy of the fills, a decision was made to conduct X-ray diagnostics in the nearest facility with appropriate equipment standards, which turned out to be a private hospital in Kalisz.

### Macrofaunal analyses

Macrofaunal fragments were discovered in eight cinerary urns, within various exploration levels/layers. A total of 17 macrofaunal fragments were identified. In burial 853, two humerus bones of a modern small mammal were discovered. The remaining 15 preserved fragments consisted of elements of chitinous exoskeletons of insects at various stages of development–four larvae and four adult forms were identified (Fig 5). The entomological material was examined using a Nikon SMZ-800 binocular microscope and a Nikon Eclipse E600 phase-contrast microscope. Taxonomic determinations were made on the basis of morphological features and entomological comparative collections. All remains were photographed using a Nikon D5100 coupled with Nikon SMZ-800. Image stacks were processed using free software CombineZM 1.0.0 [77].

The state of preservation of the discovered fragments of entomofauna was extremely varied, and most of them morphologically appeared to represent modern remains; they were fragmented but shiny and contaminated only with grains of sand that could be easily removed with a thin brush. The fragments of the insect discovered in the urn from burial 506 were covered with something resembling a crust firmly adhering to the preserved elements of the cuticle. In order to determine the species of the preserved fragments, it was decided that the left elytron will be mechanically cleaned in a petri dish using distilled water and a cotton swab. Thanks to this procedure, it was possible to recognize the structure and diagnostic elements enabling the determination of this insect down to the species level, namely, *Geotrupes stercorarius*, more commonly known as the dung beetle.

The remains of the *Geotrupes stercorarius* beetle imago were discovered above the cremated remains of a newborn within layer 3 (bottom one). Many bronze ornaments were noted in the ceiling of this layer, among them a twisted bracelet, a necklace composed of twists of "salta leone" type made from flat strips, and an ornament described by the archaeologists as an earring consisting of rings and an oval pendant (Fig 2A). The discovery of the remains of a dung beetle in the bottom layer of the urn may, of course, be accidental, however, the species designation, the morphology of the remains and the location in the urn among bronze ornaments led to a reflection on the intentional use of the beetle as an element of jewellery. Such a case would not be an isolated one, as both ethnographic [78] and archaeological [79] studies confirm cases of using insects as elements of jewellery or even making multi-element necklaces out of them. Some of the beetles included in the family Geotrupidae, especially the occurring within the Mediterranean basin *Scarabaeus sacer*, were of symbolic and religious significance [80]. The commercial contacts of the Lusatian culture community, especially during the Early Iron Age, with this part of the world are represented by numerous archaeological finds [27, 31]. Moreover, from the territory of Poland, from the cemetery of the Lusatian culture in Cieszków, the grave of a 3-4-year-old child, dated to the V BA, comes the discovery of an imported Egyptian faïence amulet with the image of Ptah-Patek with a scarab on his head [81–83]. These facts prompted an in-depth analysis of the preserved fragments of *Geotrupes stercorarius* as well as of the remaining macrofauna identified in the urns.

Both preserved dung beetle elytra discovered in urn from burial 506: the uncleaned right one (sample A) and the cleaned left one (sample B) were subjected to imaging and elemental composition analyses under the Hitachi S-3400N scanning electron microscope with the Thermo Scientific Ultra Dry EDS detector (SEM-EDS) at the Electron Microscopy Laboratory at the Faculty of Chemistry, University of Wrocław. Additionally, one modern beetle *Geotrupes stercorarius* from the collection of the Department of Invertebrate Biology, Evolution and Conservation at the Faculty of Biological Sciences of the University of Wrocław was used as a reference sample (sample C). Moreover, the piece of jewellery next to which the remains of the dungy beetle were found was also subject to SEM imaging.

### Computed tomography (CT) research

For the non-invasive studies, 19 best-preserved vessels were selected whose internal structure (fills) were not mechanically disturbed. These studies were undertaken in order to visualize the internal stratigraphy of the fill layers, to locate and virtually identify bone fragments and archaeological artefacts, such as metal items and other pottery vessels, as well as to identify the activity of soil fauna and flora. From the spectrum of many possibilities offered by CT scanning, this work focuses primarily on the possibilities of observing and identifying bioturbation processes, correlating them with specific species of animals and assessing their impact on the primary stratigraphy of the urn fills.

The vessels were secured directly at the excavation site during the archaeological works. About 40 elastic bandages were used in order to tightly wrap the vessels, each of them 10 or 12 cm wide. This was primarily to protect the ceramic vessels against further cracking and disintegration that would also affect the loose material constituting their fills. The selected vessels were subject to tomographic tests at Kaliska Agencja Medyczna MEDIX Sp. z o. o. in Kalisz using a Philips Diamond Select Brilliance CT 16-slice scanner. The thickness of the scanning layer was 0.6–1.5 mm, pitch 1.0, average lamp rotation time of 1.0s, lamp voltage 120kV, the current intensity [mAs] was variable (modulated according to the volume of the urns). The image matrix was 512x512. The images were reconstructed as required in cross-sections and frontal sections with the resolution ranging from 0.6 to 10 mm. The CT images were visualized with RadiAnt DICOM Viewer 2020.2. Image post-processing techniques included the following two- and three-dimensional reconstructions: MPR—multiplanar reformated reconstruction, MIP—maximum intensity projection, MinIP—minimum intensity projection, VR—volume rendering.

## Results

### Results of the analyses of macrofaunal remains

In the course of the research, taxonomic determinations were made regarding all discovered insect remains. A detailed list of the found remains is included in Table 1. Out of eight insects, three were identified to the species level and the rest to the genus or family level. The remains of four larvae were found in three urns, two of which were desiccated larvae of insects from the family Geotrupidae (Insecta: Coleoptera) found in the bottom layers. Two larvae of the genus *Harpalus* sp. (Insecta: Coleoptera: Carabidae) were discovered in one urn among human bones halfway down the height of the urn. The remaining entomological remains should be associated with adult forms of insects.

**Table 1:** Identified macrofaunal remains from various exploration levels (layers) within the urns from the Lusatian culture cemetery in Wtórek, Greater Poland, Poland.

Grave number	Vessels no./Layer no.	Description of macrofaunal remains	Taxon
**504**	A/layer 3	dried larva	Geotrupidae (Insecta: Coleoptera)
**506**	A/layer 3	fragments of an adult insect (imago): pronotum, scutellum, right and left elytra	*Geotrupes stercorarius* (Insecta: Coleoptera: Geotrupidae)
**507** *	A/layer 5	dried larva	Geotrupidae (Insecta: Coleoptera)
**533**	A/layer 3	fragment of an adult insect (imago)	Staphylinidae (Insecta: Coleoptera)
**542**	A/layer 1	head of imago	*Harpalus flavescens* (Insecta: Coleoptera: Carabidae)
**599** *	A/layer 2	fragments of two larvae: 1. head, 2. mandibula and frons	*Harpalus* sp. (Insecta: Coleoptera: Carabidae)
**615**	A/layer 1	fragments of an adult insect (imago): head, pronotum, right and left elytra	*Anisodactylus nemorivagus* (Insecta: Coleoptera: Carabidae)
**853**	A/layer 3	2 humerus (right and left) of a juvenile (proximal epiphyses not adhered)	*Microtus* sp. (Mammalia: Rodentia: Cricetidae)

* the vessels were subjected to tomographic examination prior to exploration

Among the analysed insect remains the presence of beetles from the families Staphylinidae and Carabidae was confirmed. Most species of Staphylinidae (rove beetles) are active predators that eat eggs, larvae and pupae of other insects and other small invertebrates [84]. Similarly, most representatives of the Carabidae family (ground beetles) are predators attacking, for example, larvae of other insects. The remains of insects discovered in three urns were assigned to this family. In two cases, the imago and remains of two larvae of the genus *Harpalus* sp. were identified, while in one—the imago of *Anisodactylus nemorivagus*. Larvae of ground beetles of the genus *Harpalus* sp. and *Anisodactylus* sp. are polyphagic, they live in the soil in which they dig tunnels [84]. The exact ecological requirements of *Anisodactylus nemorivagus* are poorly understood due to their rare occurrence in Poland [84]. Larvae of *Harpalus* sp. often prefer plant food, and their vertical burrows are filled with seeds above the terminal cell in which they live [85–87].

On the other hand, species belonging to the family Geotrupidae are mostly herbivorous and feed on decaying plant debris, humus and sap, as well as living plant tissues. Some species are scavengerous and coprophagous, such as, for instance, *Getrupes stercorarius* whose remains were discovered in the urn from grave 506. Moreover, adults of this species show quite advanced concern for their offspring. They dig in soil vertical corridor under excrements of herbivorous mammals (e.g. horses, cows) with side branches extending from it that end with nest chambers. Often the system of these corridors forms complex nests [84, 88, 89]. In the nest chambers, fertilizer (faeces) is stored, on which the female then lays eggs. Larval development takes about 12–13 months. The new generation appears from late June to early October. It can be assumed with a high probability that the dried larvae of the grub type found in the examined material (burials 504 and 507) belong to the species of the genus *Geotrupes* sp.

**Fig 6:**
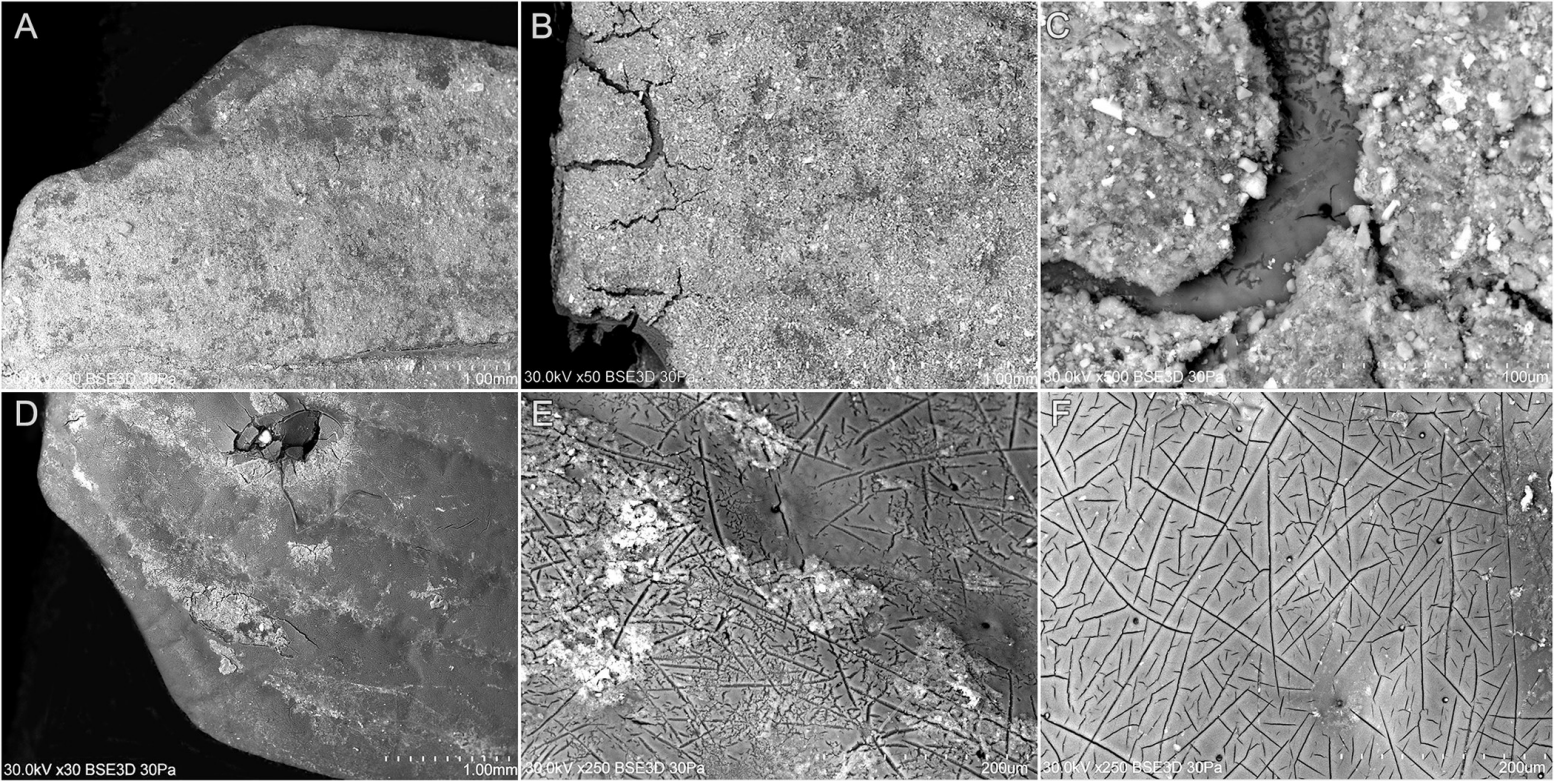
SEM imaging of *Geotrupes stercorarius* insect remains from the urn originating from burial 506. Right elytron covered with sediment (A, B, C), left elytron with sediment cleaned off (D, E) and the surface structure of the elytron of the modern reference insect (F).

Due to the enigmatic context of the discovery of the remains of the adult insect *Getrupes stercorarius* in the urn from burial 506, it was subject to scanning and elemental composition analysis using SEM-EDS. The SEM-EDS studies carried out on three samples revealed significant morphological differences between the fragments of the insect from the archaeological context (samples A and B) and the modern reference insect (sample C) (Fig 6). Scanning of the uncleaned right elytron revealed that the identified insect remains were covered with a tightly adhering layer of pressed granular sediment, which had cracked in some places (Fig 6A–6C). On the surface of the left elytron, which was mechanically cleaned, there were dendritic pits and oval dimples (Fig 6D and 6E) as compared to the elytron surface of the reference insect (Fig 6F), which proves the existence of a chitin degradation process, and, from the archaeological perspective, that the insect was in the urn for a long time.

**Fig 7:**
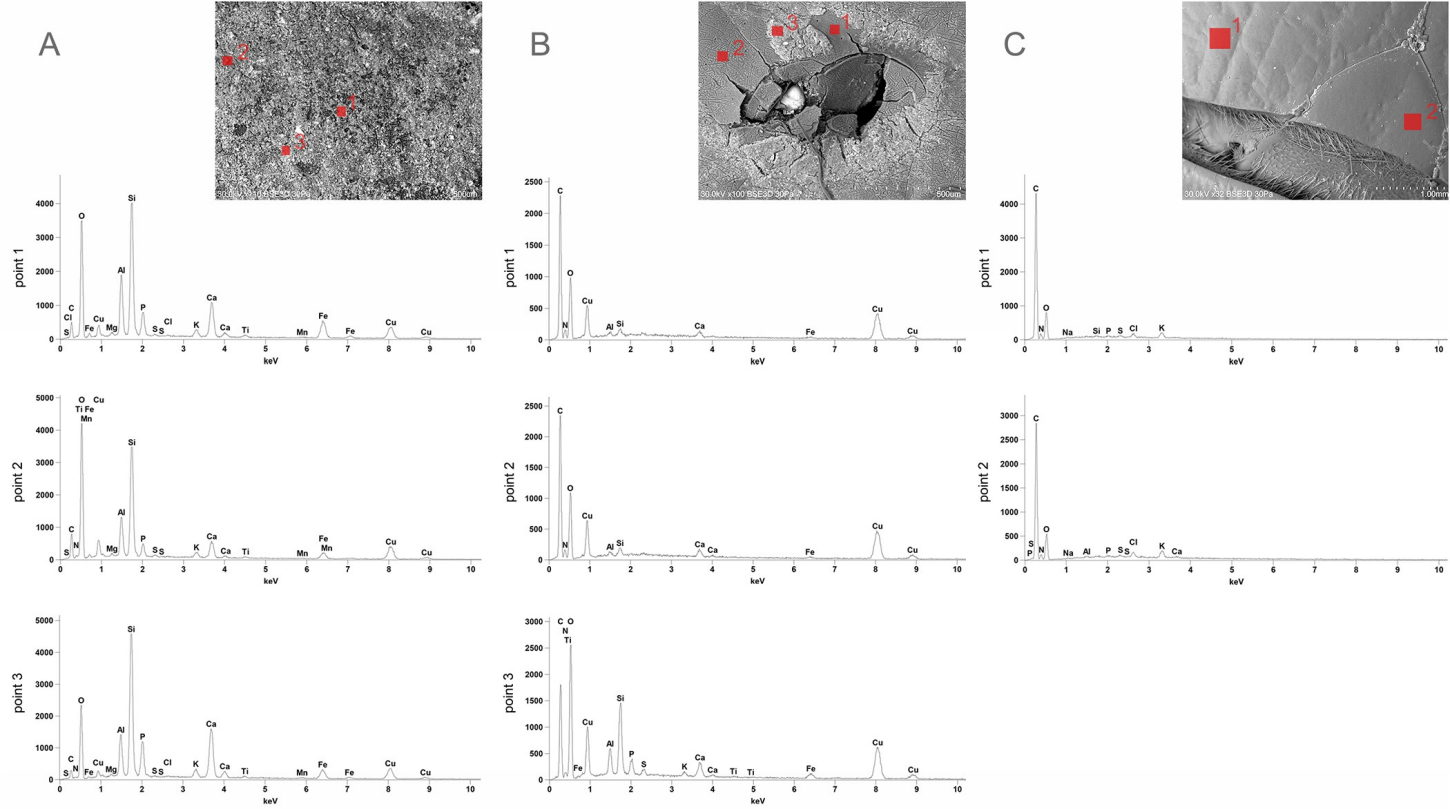
The results of the SEM-EDS elemental composition tests of two samples from *Geotrupes stercorarius* beetle coming from the urn from burial 506 (samples A and B) and the reference sample of a modern insect. Sample A consists of right elytron covered with sediment, sample B consists of left elytron with cleaned off sediment, and sample C represents the modern insect.

The analysis of the elemental composition also showed significant differences between the tested samples (Fig 7). The right elytron, from which the sediment was not removed, at all three measuring points, showed the presence of a large amount of silicon (Si), oxygen (O), aluminium (Al) and calcium (Ca), as well as small amounts of various metals (Fe, Mn, Mg). Probably the layer of the sediment was composed of loamy minerals present in the soil and hydroxyapatite originating from burnt human bones. Perhaps, this layer was deposited on the insect fragments as the result of a sedimentation process taking place within the urn. Macroscopically, similar sediment was present on the surface of some of the burnt human bones.

In the SEM-EDS analyses, copper (Cu) was the element characteristic for both archaeological samples (samples A and B). Larger amounts of this element were found in the sample with the sediment cleaned off, which probably should be interpreted as a part of the process of mineralization (pre-mineralization) of chitin with the metals coming from the corrosion of bronze artefacts (Table 2). On this basis, however, it is difficult to assess the rate of chitin petrifaction, the more so as this process depends not so much on the time of its occurrence, but on the nature of the natural environment [90].

**Table 2:** The results of the SEM-EDS elemental composition analysis measured at individual points on samples A and B (remains of *Geotrupes stercorarius* from the urn from burial 506) and sample C (modern *Geotrupes Stercorarius*). The measurement points for each sample correspond to the individual plots shown in Fig 6.

		Weight % (atom %)															
		C-K	N-K	O-K	Mg-K	Na-K	Al-K	Si-K	P-K	S-K	Cl-K	K-K	Ca-K	Ti-K	Mn-K	Fe-K	Cu-K
**Sample A—right uncleaned elytron**	point 1	8.90 (14.47)		51.23 (62.53)	0.39 (0.32)		6.60 (4.78)	14.41 (10.02)	3.05 (1.92)	0.18 (0.11)	0.08 (0.04)	0.81 (0.40)	4.24 (2.07)	0.35 (0.14)	0.22 (0.08)	4.49 (1.57)	5.06 (1.55)
	point 2	12.16 (17.89)	4.46 (5.63)	56.55 (62.47)	0.41 (0.30)		4.19 (2.75)	11.00 (6.92)	1.37 (0.78)	0.19 (0.10)		0.52 (0.23)	1.86 (0.82)	0.24 (0.09)	0.11 (0.04)	1.49 (0.47)	5.45 (1.52)
	point 3	7.07 (11.83)	2.86 (4.11)	45.86 (57.59)	0.37 (0.31)		5.08 (3.78)	16.75 (11.98)	5.12 (3.32)	0.11 (0.07)	0.13 (0.07)	1.03 (0.53)	7.14 (3.58)	0.26 (0.11)	0.19 (0.07)	2.75 (0.99)	5.29 (1.67)
**Sample B—left cleaned elytron**	point 1	32.14 (41.28)	14.16 (15.60)	41.12 (39.65)			0.37 (0.21)	0.67 (0.37)					0.53 (0.20)			0.30 (0.08)	10.71 (2.60)
	point 2	31.88 (41.18)	13.91 (15.41)	40.92 (39.69)			0.40 (0.23)	0.73 (0.40)					0.70 (0.27)			0.31 (0.09)	11.14 (2.72)
	point 3	23.60 (32.45)	7.15 (8.43)	49.40 (51.01)			1.97 (1.20)	4.92 (2.90)	1.08 (0.57)	0.38 (0.19)		0.26 (0.11)	1.11 (0.46)	0.08 (0.03)		0.82 (0.24)	9.23 (2.40)
**Sample C—modern reference insect**	point 1	40.89 (47.26)	18.89 (18.73)	38.21 (33.16)		0.30 (0.18)		0.16 (0.08)	0.14 (0.06)	0.18 (0.08)	0.45 (0.17)	0.78 (0.28)					
	point 2	40.24 (46.78)	18.47 (18.41)	38.61 (33.70)		0.26 (0.16)	0.20 (0.10)		0.22 (0.10)	0.21 (0.09)	0.6 (0.23)	1.02 (0.36)	0.19 (0.07)				

Most of the elements identified in the archaeological samples were not present in the reference sample (sample C). In the spectrum of the reference sample, contaminants of the chitin surface consisting of Cl, K, and Na elements are present (Fig 7C; Table 2). They come from chemical substances used to kill insects with chloroform (CHCl _3_), chemical treatment (bath in NaOH) and preparation (NaCl and KCl). The presence of these substances did not go unnoticed in the SEM-EDS analyses of the reference sample.

**Fig 8:**
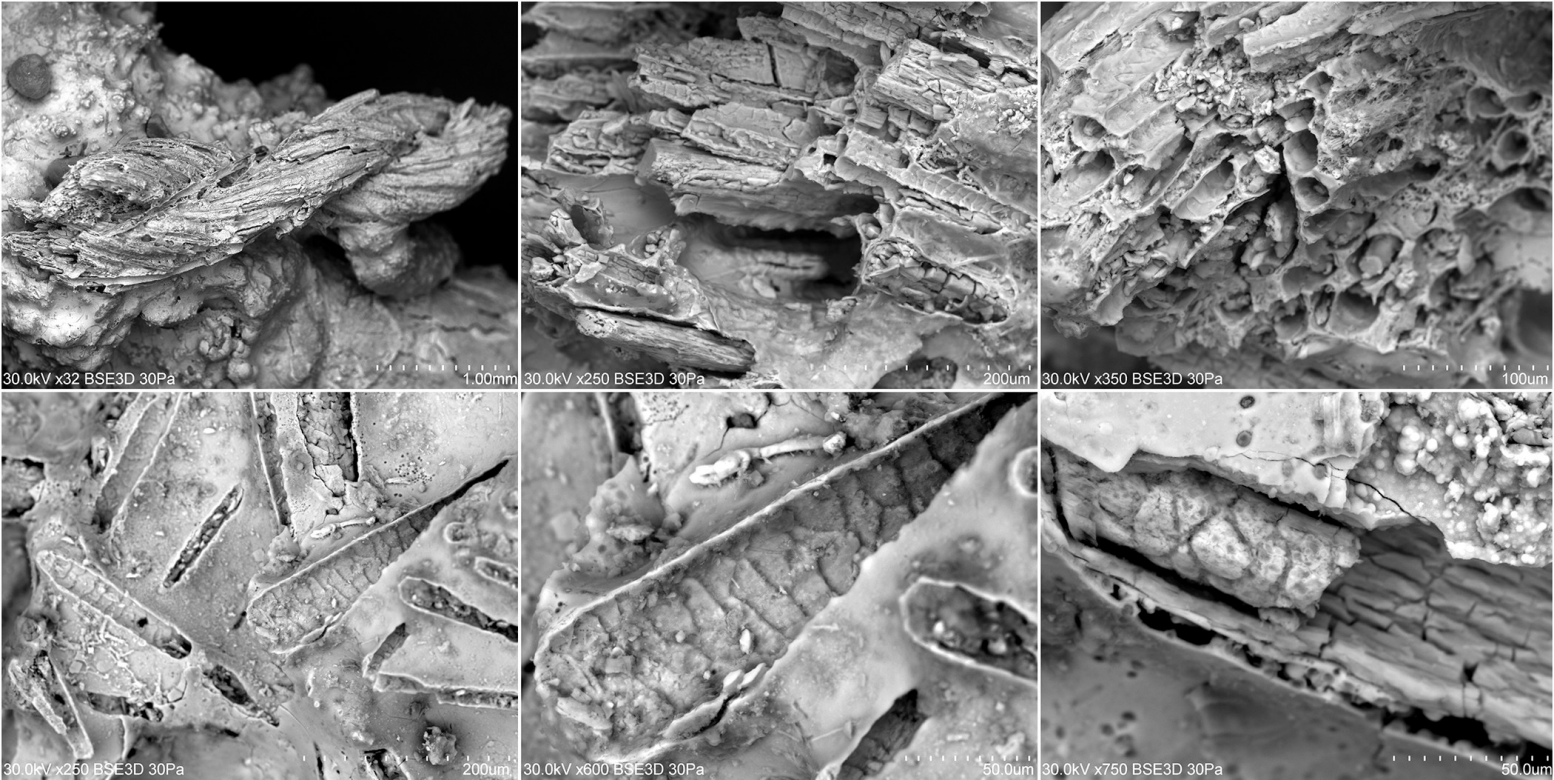
The textile pseudomorph of the woollen cloth from the urn from burial 506. Identified in SEM imaging woollen fabric fragments were preserved as textile pseudomorph located on the underside of one of the jewellery pieces that had been placed over the cremated bones of a newborn.

In the SEM imaging of one of the "earring" rings, under which the remains of *Getrupes stercorarius* were located, the presence of a fragmentarily preserved fabric in the form of a textile pseudomorph was discovered (Fig 8). For the production of the textile, sheep wool was used as the raw material, the fragments of which were present on the underside of the ornament. However, it cannot be ruled out that originally the fabric was, for example, a bag in which the jewellery pieces had been kept. In the same way, it cannot be determined whether the fabric was not a kind of protection for the cremated bones of a newborn, which, for example, could have been covered with a cloth. It is certain, however, that in the urn there were favourable conditions for petrification, in the process of which organic substances could "survive" thanks to their replacement with inorganic compounds present in bronze jewellery.

The remains of a modern vertebrate discovered in the bottom layer of the urn from burial 853 were identified as belonging to the voles genus *Microtus* sp. These small rodents dig holes in the soil where they live and collect, among others, food such as tubers, rhizomes, cereal seeds, etc. Their bioturbation activity in a general environmental context is not significant [91], however, in the archaeological context it contributes to disturbance of stratigraphy and relocation of human bones as well as to leaving pseudo-pathological traces on them [92].

### Results of the CT analyses

**Fig 9:**
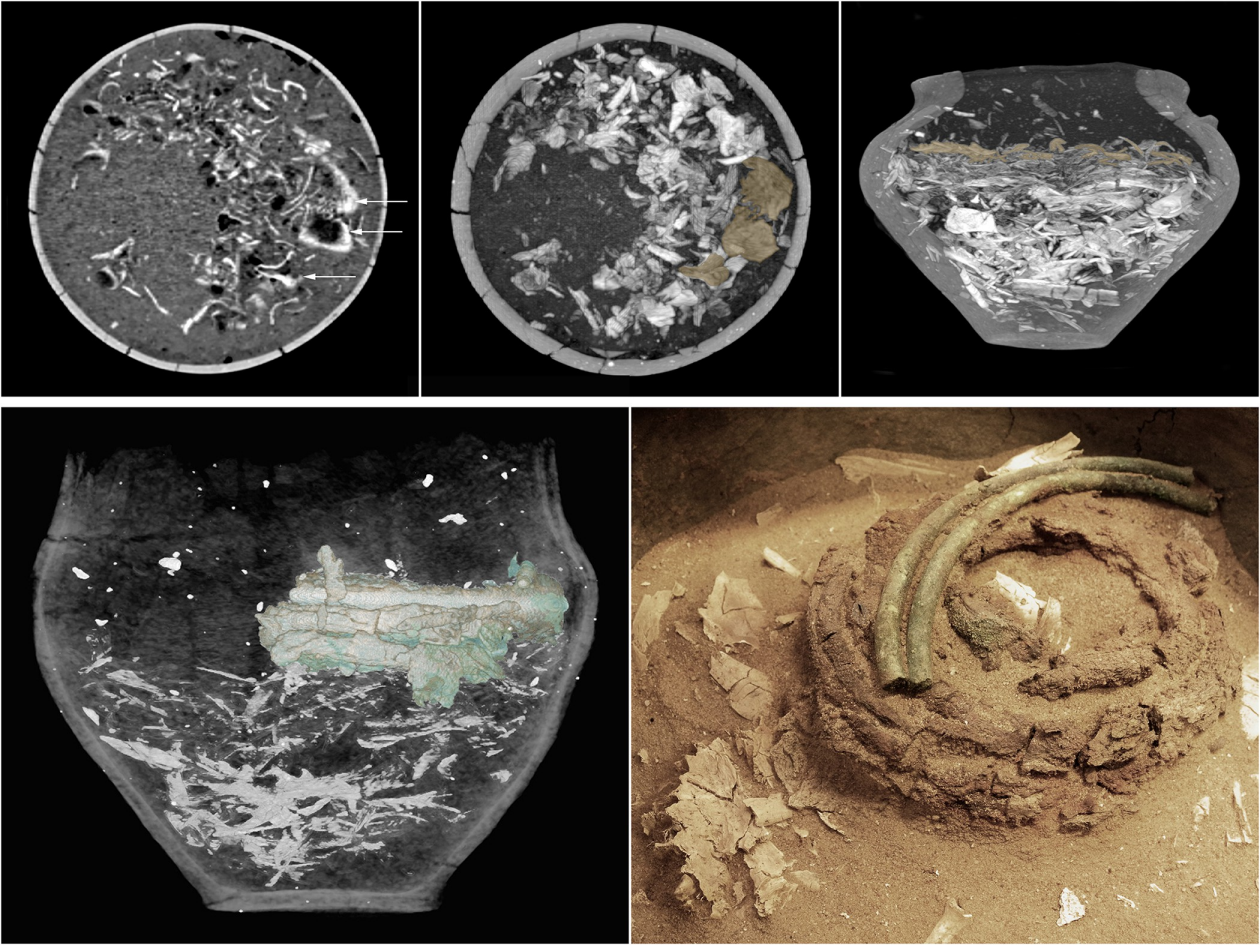
The results of CT imaging of the fills of the urn from graves 557 and 528. In the upper row, fragments of the skull vault bones are visible in the upper part of the fill of the urn from grave 557; the arrows and the brown colour mark the identifiable fragments of the parietal bones and the right zygomatic bone. In the lower row, the urn from grave 528 and the visualization of metal objects in MIP reconstruction and their actual appearance during the exploration.

**Fig 10:**
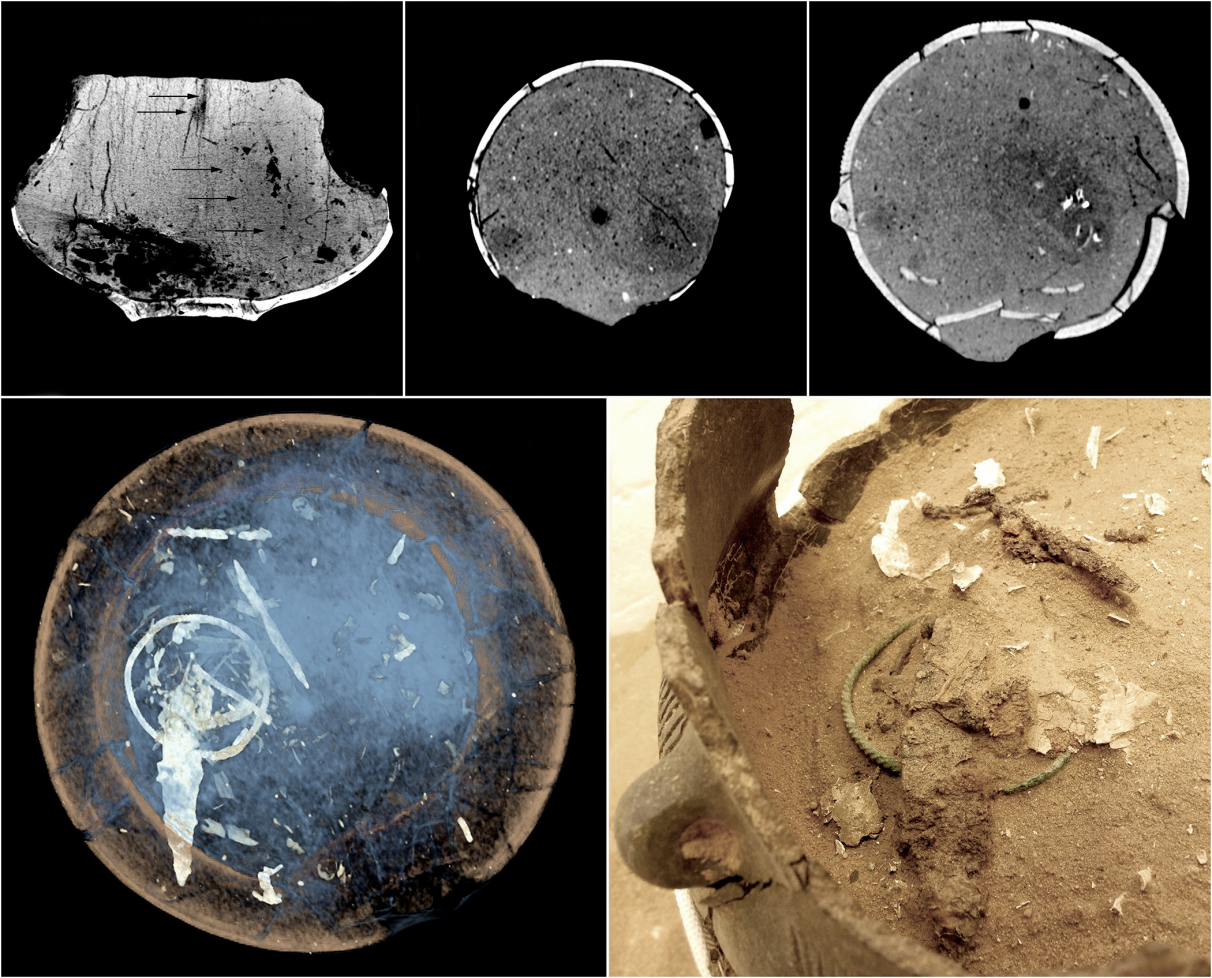
The results of CT imaging of the fill of the urn from grave 507. In the top row, MinIP reconstructions of the urn fill; the arrows mark a fragment of the disappearing corridor and the nest chamber of a beetle of the genus *Geotrupes* sp. as well as the disappearing rodent burrow. At the bottom, the VR reconstruction of the arrangement and location of metal items in the urn and their actual appearance during the exploration.

The CT examinations of 19 vessels showed that two of them were additional vessels in which no osteological material was found, while the remaining vessels were cinerary urns with burnt human bones. Grave goods recorded in the urns in the form of bronze and iron jewellery and pottery vessels were placed over human bones in layers closest to the vessels rims (Figs 9, 10, and S1). The burnt human remains were arranged in layers, the upper layer of which most often consisted of the bones of the skull (Fig 9).

Location and visualization of traces of soil fauna activity require a deliberate search for them among the data obtained through CT scanning. They are not always visible in CT slices and depending on the density of soil and artifacts in vessels and the software used for data post-processing, we are forced to test various types of 3D visualizations. Some traces are much better visible after cutting off the maximum values of radiological density (HU), e.g. in MinIP, while others could be visualized much better using different VR variants.

**Fig 11:**
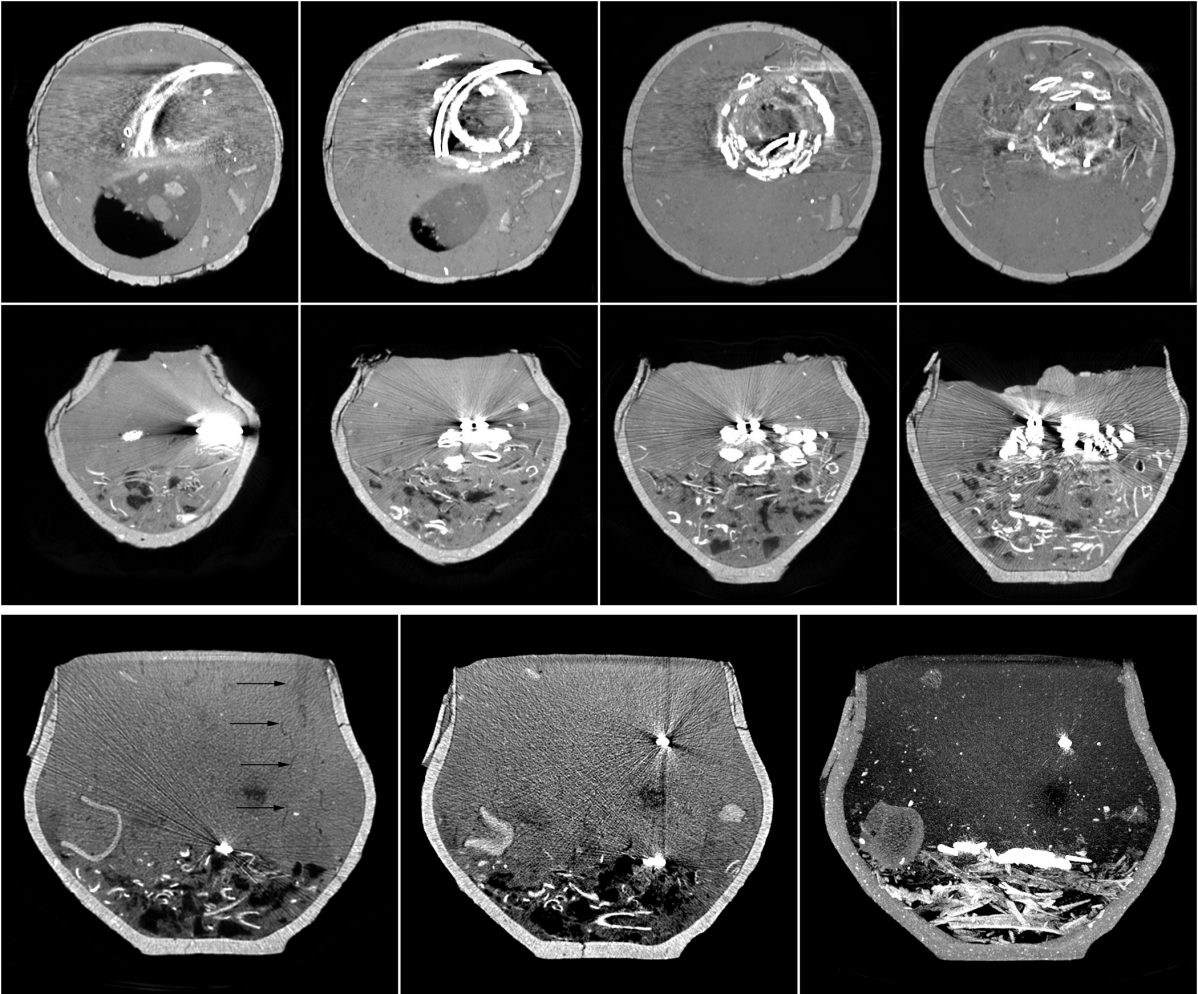
Examples of metal-related artifacts in CT slices of the urns from graves 528 and 548. The upper two rows show the fill of the urn from grave 528, in which the presence of iron ornaments made it impossible to visualize traces of soil fauna activity. Artefacts are visible in the form of bright and dark streaks interfering with the reading of the imaging itself. The lower row shows artefacts of a similar type that were recorded in the CT imaging of the fill of urn 548. In this case, it was possible to identify a disappearing rodent burrow (black arrows), in which there is now a piece of metal that has been displaced significantly above its original position.

In the case of five vessels, no bioturbative activity was observed. Only the presence of ingrown plant roots was recorded in them. In the case of one of the examined urns, the observation of bioturbative activity turned out to be impossible due to significant CT beam hardening manifesting itself in the form of streaking and ring artefacts resulting from the accumulation of a large number of iron ornaments (Fig 11). Metal-related artifacts appear in the form of bright and dark streaks and are mainly caused by two phenomena: beam hardening and photon starvation. The main disadvantage of CT imaging is the occurrence of metal artifacts, which degrade image quality and hinder soil disturbances evaluation.

**Fig 12:**
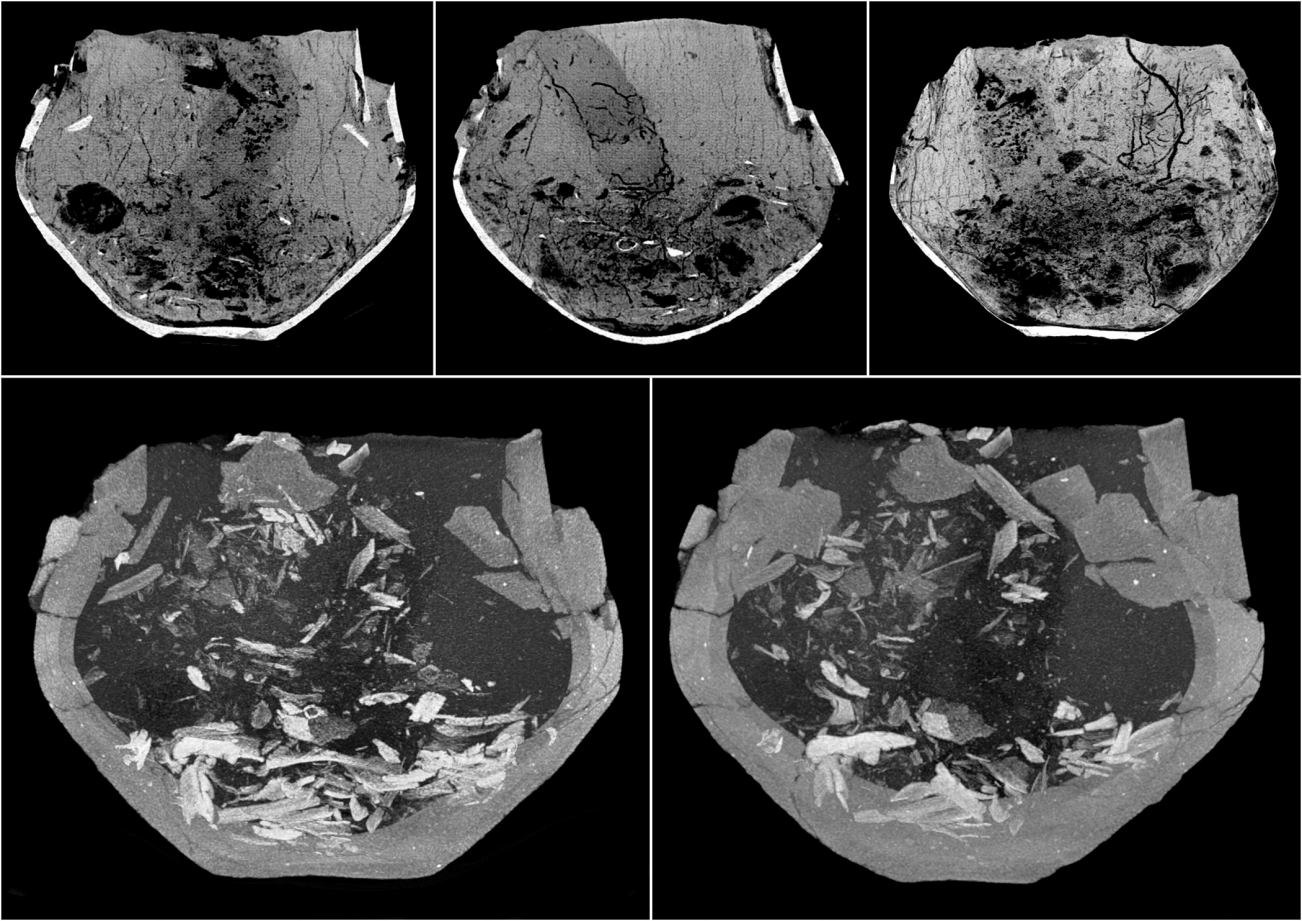
The CT imaging of the fill of the urn from grave 877. The top row shows the MinIP reconstruction of the urn fill with a visible system of burrows and ingrown plant roots. At the bottom, the MIP reconstruction of the displaced upwards and mixed burnt bones.

The presence of various traces of soil macrofaunal activity was identified in the remaining 12 cinerary urns and one additional vessel. In four urns, old and buried rodent burrows of rodents or other burrowing animals were found (Table 3). In all these cases, the burnt bones were displaced into the higher layers, as evidenced by the CT imaging (Figs 10 and 12). The specificity of the sandy soil did not allow for the observation of this process during exploration, as the differences visualized on the CT reconstructions resulted from both differences in density and compaction of individual layers, as well as different types of materials forming the fills. A "rodent passage" left a trace in the form of loosening (aeration) of a part of the urn fill, which was an integral part of the burrow that was very clearly visible in the MinIP reconstructions. At the same time, by loosening the soil the rodents contributed to a greater plant penetration and increased root ingrowth in these areas (Fig 12). In the urn from grave 877, a system of burrows reaching the bottom of the vessel was visualized. Burnt human bones originally lying below the maximum diameter of the vessel belly were displaced above this level and significantly fragmented. The original layers and the intentionality of their deposition are now difficult to reconstruct. It should be emphasized that we obtained this information only thanks to the CT visualization, as the burrows did not manifest themselves in the ceilings of individual exploration layers.

**Table 3:** Characteristics of the traces of macrofaunal activity observed in the CT scans and taxonomic identification of particular bioturbations.

Grave number	Vessel number / function	Description of the traces of macrofaunal activity observed in CT imaging	Taxonomic identification of the bioturbator	Comments
**485**	A/urn	1. vertical, short corridor; 2. buried nest of *Geotrupes* sp.	1. Burrow of *Harpalus* sp.; 2. beetles of the genus *Geortupes* sp.	beam hardening
**507**	A/urn	1. disappearing rodent burrow; 2. disappearing nest of *Geotrupes* sp.	1. small rodents 2. beetles of the genus *Geortupes* sp.	upwardly displaced fragment of a metal item and burnt human bones, including fragments of the petrous part of the temporal bone; beam hardening
**526**	A/urn	fragment of the disappearing corridor with the nest chamber of *Geotrupes* sp.	beetles of the genus *Geortupes* sp.	beam hardening
**548**	A/urn	1. fragment of a buried nest of *Geotrupes* sp.; 2. disappearing rodent burrow	1. beetles of the genus *Geotrupes* sp.; 2. small rodents	upwardly displaced fragments of burnt bones with a fragment of a metal item; beam hardening
**570**	A/urn	short vertical corridor	possibly a corridor of an insect of the genus *Harpalus* sp.	beam hardening
**574**	A/urn	short vertical corridor	possibly a corridor of an insect of the genus *Harpalus* sp.	
**599**	A/urn	1. short, vertical corridor; 2. two buried channels	1. burrow of *Harpalus* sp.; 2. unidentified taxon	caryopses of *Setaria* sp. present in the vertical corridor; beam hardening
**600**	A/ additional vessel	1. earthworm corridor; 2. short corridor ended with a chamber at the urn rim	1. earthworm tunnel 2. aestivation chamber of an earthworm	Additional vessel with no cremated bones present
**628**	A/urn	probably a buried nest of *Geotrupes* sp.	beetles of the genus *Geortupes* sp.	
**632**	B/urn	1. vertical corridor; 2. corridor along the vessel wall; 3. corridor with nest chambers—nest	beetle nests of the genus *Geortupes* sp.	
**745**	A/urn	disappearing rodent burrows	small rodents	burnt bones displaced upwards; beam hardening
**877**	A/urn	rodent burrows	small rodents	Numerous burnt bones displaced upwards; numerous plant roots ingrowth into the mixed soil of the burrows
**897**	A/urn	short vertical corridor	possibly a corridor of an insect of the genus *Harpalus* sp.	

The chances of visualizing bioturbations decrease with time since their creation. Freshly formed traces of activity are much more visible and easier to visualize (Fig 12) than their older counterparts (Fig 10). The type of substrate is probably responsible for the chances of their preservation. In sandy soils, sorting processes take place quite quickly due to high water permeability of the substrate, therefore many bioturbation processes may remain unnoticed.

**Fig 13:**
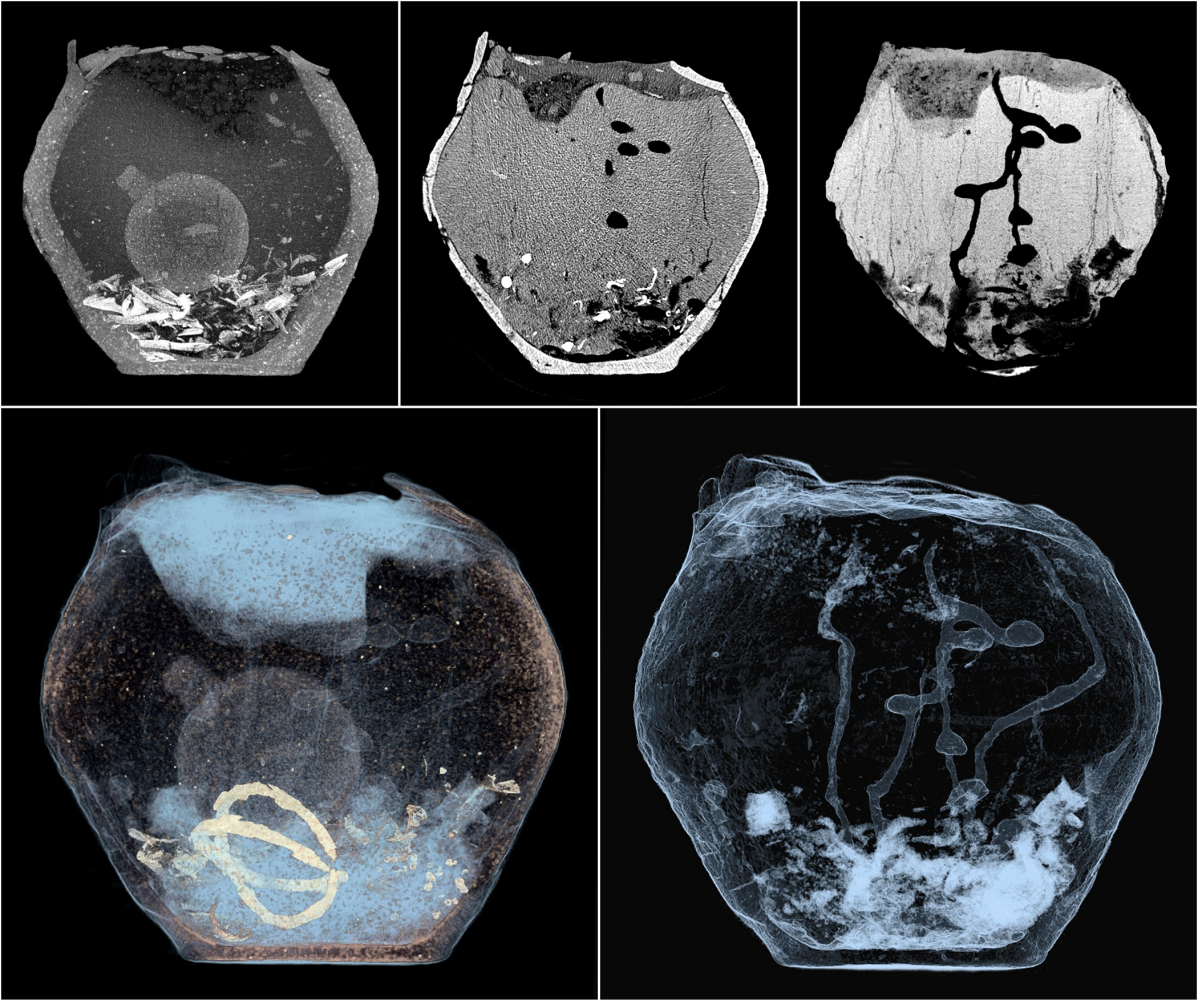
The imaging the urn B from grave 632 in order to accentuate the complexity of the elements of the fill. The MIP reconstruction shows an additional vessel placed over the burnt bones, while in the CT slices individual nest chambers of *Geotrupes stercorarius* are visible, and in the MinIP reconstruction, a part of the dung beetle nest is visualized (top row). At the bottom—the VR reconstruction, which contains all the components of the fill, including metal ornaments, and the VR reconstruction highlighting the complexity and state of preservation of the *G*. *stercorarius* nest filling up the urn.

**Fig 14:**
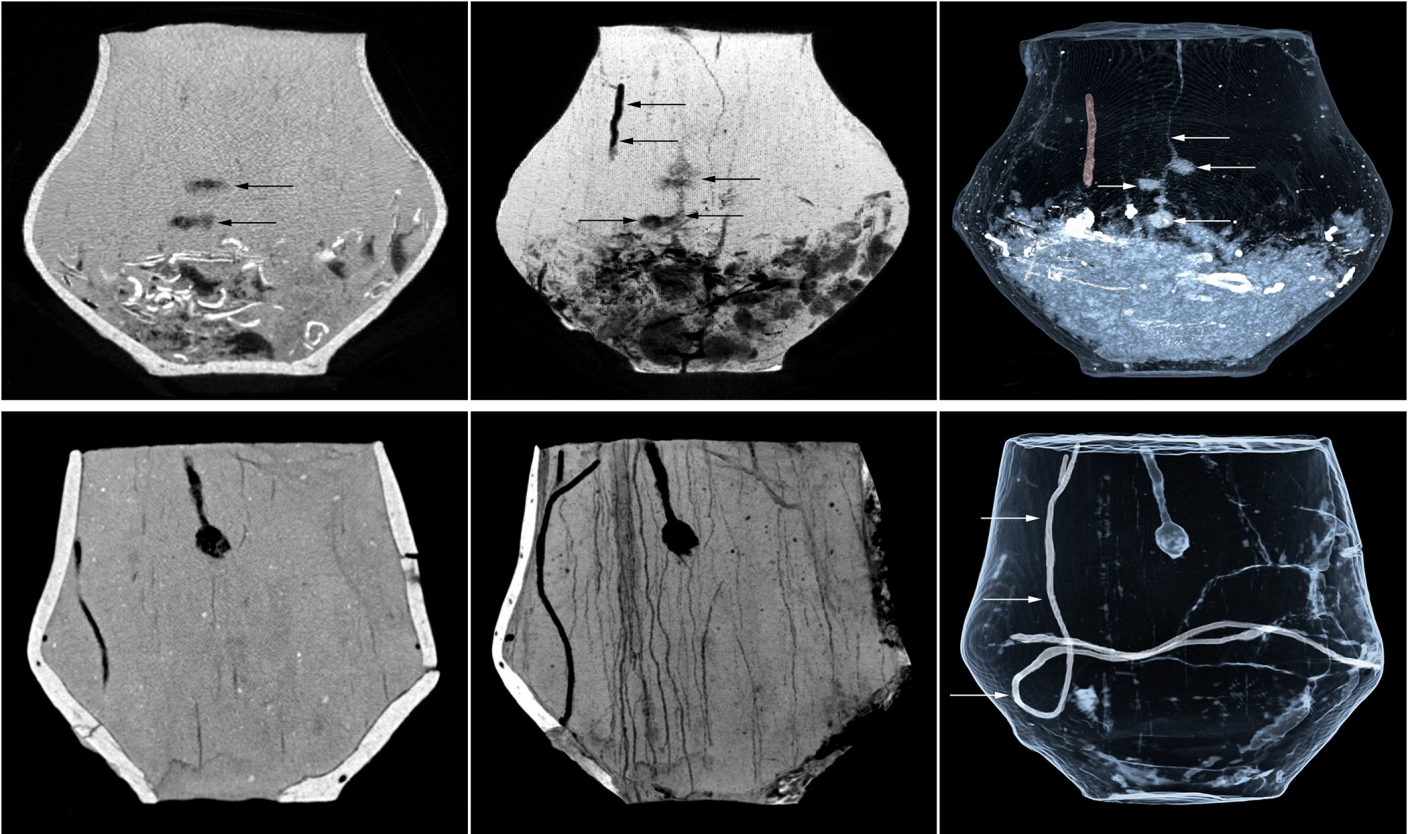
The CT imaging of the fill of the urn from grave 485 and the fill of the additional vessel from grave 600. At the top, the visualization of the urn fill from grave 485 with a preserved corridor of an insect of the genus *Harpalus* sp. and a disappearing nest of a beetle of the genus *Geotrupes* sp. At the bottom, the imaging of the interior of the additional vessel from grave 600 with an earthworm tunnel and an aestivation chamber of a different species of earthworm marked with arrows.

The process of disappearance of traces of macrofaunal activity does not only concern traces of rodent activity. A similar type of process was identified in relation to the nests set up by *Geotrupes* sp. Generally, the occurrence of nests or parts of nests of beetles of the genus *Geotrupes* sp. was observed in six urns (Table 3). In the urn B from grave 632, perfectly visible traces of activity of *Geotrupes* sp. were discovered. In this urn, a very well-preserved entire nest of a dung beetle was discovered (Figs 13 and S2; S1 Video). Both the main vertical corridor and the branching side tunnels ending with nest chambers were empty or filled with a highly radiolucent material, and, therefore, their VR-CT visualization was not difficult. Some of the nest chambers were located at the bottom of the vessel among burnt human bones. In the remaining urns, fragments or entire nests of *Geotrupes* sp. were discovered, whose state of preservation was much worse (Figs 14 and S2). This affected their identification and visualization. Disappearing beetle nests were completely unidentifiable in the CT slices, and they manifested themselves only in the MinIP reconstructions and poorly in the VR. Nesting by insect species of the genus *Geotrupes* sp. is closely related to the occurrence of dung of herbivorous animals above the nest, i.e. in this case above the Lusatian culture urns. If these nests were to be established shortly before the start of the excavation, they would have to descend to the depth of approx. 0.8–1.0 m from the ground surface.

**Fig 15:**
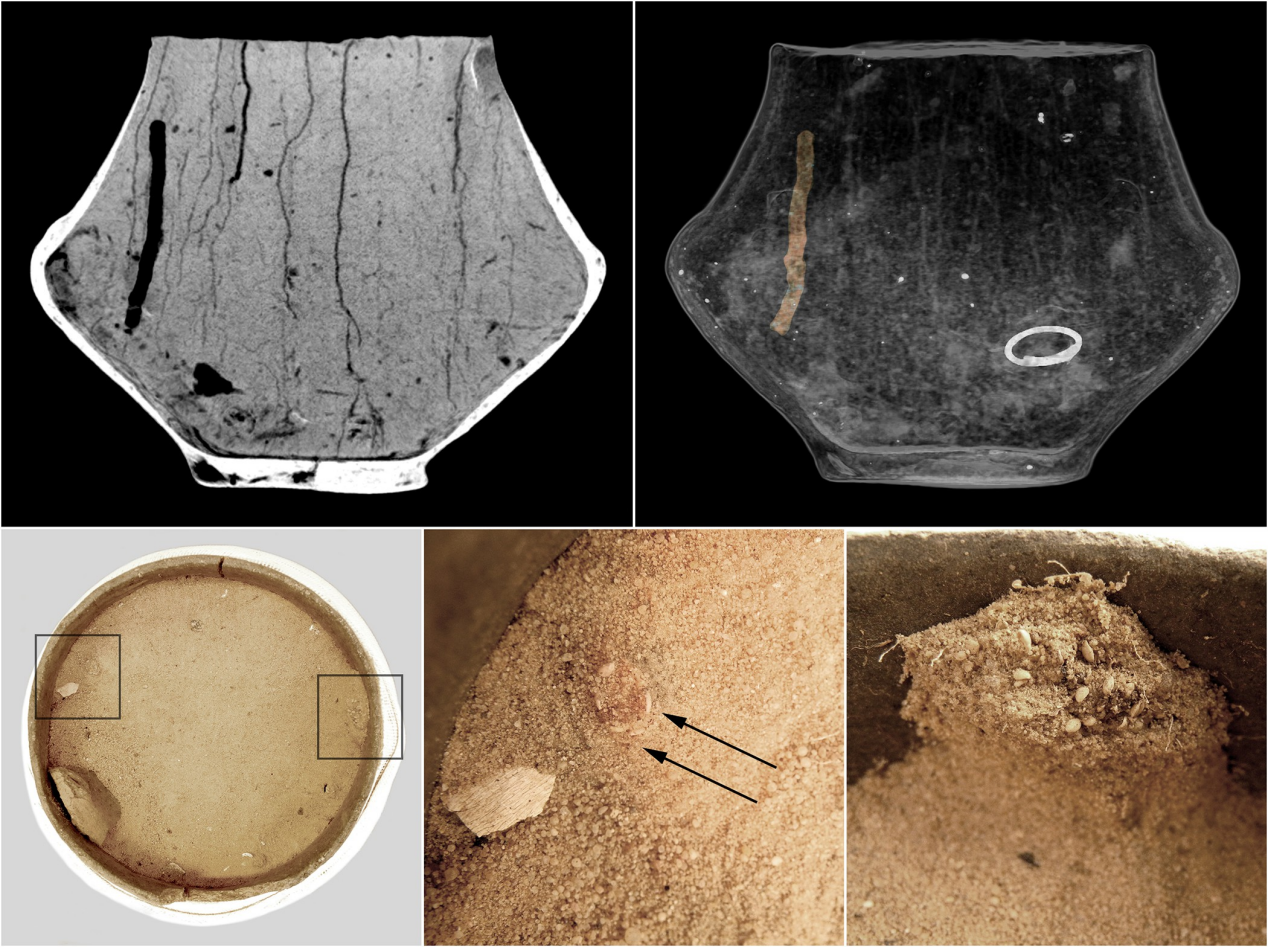
The CT imaging of the fill of the urn from grave 599. At the top, the MinIP and MIP reconstructions of the urn with a visible corridor of an insect of the genus *Harpalus* sp. At the bottom, the location of the corridor filled with *Setaria sp*. caryopses and the caryopses deposit identified during the spit exploration of the urn.

In the urn from grave 485, in which the existence of such a disappearing dung beetle nest was discovered, a short roughly 6.5 cm long vertical corridor was also revealed that started about 5 cm below the rim of the vessel (Figs 14 and S2). A similar type of structure was also found in the urn from grave 599 as well as in three other urns (Table 3). During the exploration of urn 599 in the corridor visualized in the CT imaging, numerous archaeobotanical caryopses were identified as belonging to the genus *Setaria* sp. (Figs 15 and S2). Probably, corridors of this type should be associated with beetles of the genus *Harpalus* sp., among which filling of breeding corridors with various types of seeds was frequently noted, especially with Setaria seeds, which are preferred by them most probably due to the large amount of oils they contain [86]. In general, in addition to digging corridors, these insects are capable of collecting and depositing botanical material in archaeological contexts, which undoubtedly include cinerary urns.

In the CT scans, structures resembling fragments of dung beetle nests are, in fact, aestivation chambers of earthworms, which in this way protect themselves against drought in summer. Earthworms build aestivation chambers by compacting the soil and shaping it into a sphere in which, rolled up, they go into a dormant state [93]. It may be difficult to distinguish aestivation chambers from fragments of beetle corridors ending with nesting chambers. The compacted wall of an aestivation chamber, which in CT reconstructions stands out much better from the rest of the soil background may be the element differentiating these two structures. Probably in the additional vessel from grave 600 exactly such a structure, i.e. a compacted wall, was observed (Figs 14 and S2). The chamber had the diameter of about 1.3 cm and a round outline, in contrast to the flattened nest chambers of dung beetles. Moreover, the presence of a well-preserved earthworm corridor, presumably of a different species, was recorded in this vessel. Earthworms are very common animals, especially abundant in cultivated fields. Therefore, it seems interesting that there are no traces of earthworm activity in the examined vessel fills, and the only observed traces were found in the additional vessel containing no burnt human bones.

## Discussion

Usually, bioturbation activity is studied through soil micromorphology [94, 95]. However, it is an invasive method and in the case of a conglomerate in the form of a loose sandy fill and hard cremated bones, it cannot be sampled due to the displacement of bone fragments [14]. In addition, urns often contain metal and other artefacts that could be destroyed. This method also does not provide a full picture of the ongoing bioturbations, although in some cases it may be complementary to the non-invasive studies [15].

Recognition of bioturbation processes is of key importance when interpreting the intentional arrangement of bones in urns [11–13, 96–101] or their deliberate fragmentation [8]. Identification and assignment of individual traces of bioturbative activity to specific animal taxa may also be an element of a preliminary diagnosis of contamination affecting the results of other specialist studies. In the urns where the presence of nests of *Geotrupes* sp. was demonstrated, large amounts of fatty acids can be expected from decomposing faeces with which the nest chambers were filled. Perhaps their decomposition in the immediate environment of the urn would have an impact on the results of possible chemical analyses, such as Gas Chromatographic-Mass Spectrometric analysis (GC-MS).

The conducted non-invasive studies of the urns from the cinerary cemetery at Wtórek contributed to the comprehensive examination of the urns fills. This concerns not only the elements that were also the subject of interest to other researchers [7, 16, 58] but additionally the issues that have not been so far discussed in the literature of the subject. Research limitations concerning bioarchaeological analyses of burnt human bones are, in a sense, compensated for by completely different research aspects. This is mainly due to the structure and specificity of the urns themselves and the material filling them, which constitutes a unique environment. The undoubted advantage of urns is their compactness and usually small/medium dimensions and the overall weight, which, on the one hand, allow for an easy movement of the entire vessel with its fills to a study room or laboratory, and, on the other hand, enables performing computed tomography in publicly available medical facilities.

In addition to the studies carried out on the urns, computer tomography of two additional vessels was performed as well. In their fills, despite the lack of archaeological artefacts and burnt bones, fascinating structures were discovered that were remnants of the activity of soil fauna. Interestingly, the occurrence of such structures was not observed in the examined urns. This may be due to the chemistry of the urn fill, where the large volume of bone causes significant alkalinization of the soil. Polish species of earthworms prefer environments with a neutral pH, but they live in soils with a wide pH range, mainly 5.5–8, while some also live in acidic soils. A pH above 8 is not tolerated by most earthworm species [102]. Probably the presence of burnt bones has a negative and dissuasive effect on earthworms, which in turn eliminates them as potential bioturbators of urn fills. The discovered aestivation chamber also indicates alternating periods of humidity and increased drought, which, in turn, may have further consequences when it comes to bone fragmentation and cracking due to drying.

Among the observed bioturbation processes, the activity of rodents had the greatest impact on bone fragmentation. The effects of rodents digging the burrows mainly concerned the disturbance of the stratigraphy of the primary layers and the upward movement of the bones within the vessels. Probably, in the case of soil other than sandy, their activity should be noticeable during mechanical exploration, but in the case of the cemetery at Wtórek it was not perceptible. The evidence of their direct activity was provided by the CT imaging of burrows in three urns, as well as the identification of vole bones of the genus *Microtus* sp. among burnt human bones in another urn (Tables 1, 3). Another aspect associated with digging burrows is loosening and aeration of the soil, which results in increased plant roots expansion.

The works devoted to the study of the size of burnt bone fragments do not take into account the impact of soil fauna as a bone fragmenting factor. Issues related to bone crushing and collapsing due to soil pressure are also rarely discussed [15, 59]. Another aspect affecting bone size may be their intentional breaking or crushing [8, 37, 38], but such practises were not noticed at the researched cemetery. Fragmentation of the skeleton bones may undoubtedly be increased by bioturbative factors, therefore they should be taken into account when determining the size of the bones. Another problem resulting from rodent activity is bone displacement into higher layers and possibly (though not recorded in our study) outside the urn fill. In case of increased activity of soil fauna, which is observed at some sites [103], we may have to face a misleading observation of the layered deposition of human remains above urns. Unfortunately, in such cases, if bioturbative observations are not made during archaeological excavations or CT scans are not performed, the data might be misinterpreted.

The transfer of data results to an interpretative level is often crucial for a wider understanding of funerary practices. The items deposited in the grave reflect the social position of the buried person and the relationship of the living to the deceased, but the spectrum and diversity of the so-called grave goods are immense. The most frequently observed are unique artefacts described as luxury items, such as swords, gold or amber ornaments, etc. [24, 27]. This is indispensably associated with the ability of various types of artefacts to survive in the soil for hundreds of years. Organic items preserve sporadically, therefore they are usually recorded in swamp environments [104] or in the contexts providing the possibility of petrification processes to occur, which usually are closely related to the presence of corrosive metal objects [33, 34, 105, 106]. In the case of the Lusatian culture cemeteries in Poland, the presence of, for example, fabrics [107–113] or other organic elements related to clothing [114] or ornamentation [79] was noted only at a few of them. Thus, the discovered remains of *Geotrupes stercorarius* from grave 506 became part of a more complex research question. The revealed remains of this dung beetle may indicate its intentional use as an element of jewellery. The symbolic meaning of similar beetles and the location in the urn also incline to interpret the data regarding the identified insect in such a way. The demonstrated process of pre-mineralization of the chitinous parts of the insect and the presence of the woollen fabric in the form of a preserved textile pseudomorph additionally support this interpretation. The lack of analogy, i.e. a greater number of finds of this type, is easily explained by the limited possibility of preservation of organic remains. In the case of analyses of prehistoric materials (but not exclusively), a certain research ignorance is also observed, which often excludes from the research materials that are treated as modern contaminants. This type of material includes, for example, the vole *Microtus* sp. bones examined by us, but also other macrofaunal remains (Table 1).

The demonstrated virtual presence of nests and their parts and two dried larvae (Tables 1, 3) discovered in other urns undermine the intentional use of the beetle. Unfortunately, we do not have the CT images of the urn in which the remains of the dung beetle were found. The process of building nests by dung beetles probably lasted several years, as evidenced by the recorded disappearing nests (Fig 14). The period of the highest intensity of setting up nests by dung beetles was most probably related to the use of the cemetery space as a meadow for grazing herbivorous mammals in the mid-twentieth century. The visualized nests of *Geotrupes stercorarius* usually reached to the half or even the bottom of an urn, so it cannot be ruled out that there was also a dung beetle nest in grave 506, and the adult insect had died crushed by the pressure of metal items and loose soil, for instance, while digging a nest. It is difficult to establish the speed of the chitin petrification process [90], however, the identified textiles were preserved as fully mineralized pseudomorphs. Therefore, we believe that the remains of *Geotrupes stercorarius* identified in the urn are an insertion unrelated to the funeral practices and were not placed in the urn intentionally. However, this does not change the fact that this insect belongs to the grave context and is an important element of the cognitive process of understanding the ecological variability occurring in the area of the cemetery.

From the perspective of identified burrows and remains of the insects of the genus *Harpalus* sp. and caryopses of *Setaria* sp. filling their burrows, the issue of interpretation of the found botanical remains becomes interesting. The burrows visualized in the CT imaging enabled their physical location in the urn. These structures were very sensitive to being touched by a brush, and, therefore, without a precise prior location, probably all the caryopses of *Setaria* sp. would be collected as a sample from a layer intended for archaeobotanical research, and their interpretation would not take into account the activity of insects.

As it was demonstrated, the observed variability of the ecosystems has an impact on the fill of the urn. While no structures disrupting the original stratigraphy were observed during mechanical exploration, they were visualized in the CT imaging. The fill of the urn appears to be fairly stable, but it is not unchanging. The dynamics of changes inside the urn depends on the general ecological variability, be it ecological succession or the variability of ecosystems influenced, for example, by agricultural and animal husbandry conditions. Formation of new trophic relationships at the urn level is exemplified by the identified remains of the insect from the family Staphylinidae representatives of which are actively hunting, for example, for larvae of other insects, such as grubs of the genus *Geotrupes* sp.

The research results presented in this work lead to reflection on the legitimacy and often compulsion of exploring urns and additional vessels from the cemeteries of the Lusatian culture. The occurrence of the remnants of soil fauna activity or the ingrowth of plant roots into the additional vessels may be an interesting research aspect that can also be transposed to other epochs. In the burials of many prehistoric cultures (e.g. the Funnel Beaker culture, the Corded Ware culture or the Bell Beaker culture), human remains are accompanied by vessels deposited in graves, which can also be subject to imaging in a similar way as in this study.

It should be emphasized that the data obtained from the mere anthropological analyses of the burnt bones are often very limited due to the poor state of preservation or significant fragmentation of the remains. As already mentioned, the cemeteries of the Lusatian culture are one of the most numerous archaeological features studied. Usually, in cemeteries, most of the graves are damaged to a smaller or larger extent, which eliminates the possibility of conducting non-invasive tests. Another important fact resulting from the research undertook in this work is drawing attention to the holistic approach to archaeological excavations and treating the archaeological site as a "transforming structure". Of course, the diversity and enormity of information obtained in this way can be overwhelming, but by exploring archaeological features we actually destroy them irretrievably. Therefore, the use of non-invasive and non-destructive methods should be a priority during the undertaken research activities.

## Supporting information

S1 Video CT data visualization of the urn B from the grave 632. (MP4)

S1 Fig The VR reconstructions of the arrangement and location of metal items in the urn from grave 507 and their actual appearance during the exploration. (TIF)

S2 Fig Visualisation of the urn and additional vessel fills (urn B from grave 632 (A), urn from grave 485 (B), urn from grave 599 (C), additional vessel from grave 600 (D)) with a preserved nests of the genus *Geotrupes* sp. (1), burrows of the genus *Harpalus* sp. (2), aestivation chamber (3) and tunnels of earthworms (4).
(TIF)
