# Supplementary figures and images for: Let’s make a mess, maybe no one will notice. The impact of bioturbation activity on the urn fill condition
Source: PLoS One. 2022 Sep 2;17(9):e0274068. doi: 10.1371/journal.pone.0274068 (PMC9439216; doi:10.1371/journal.pone.0274068)

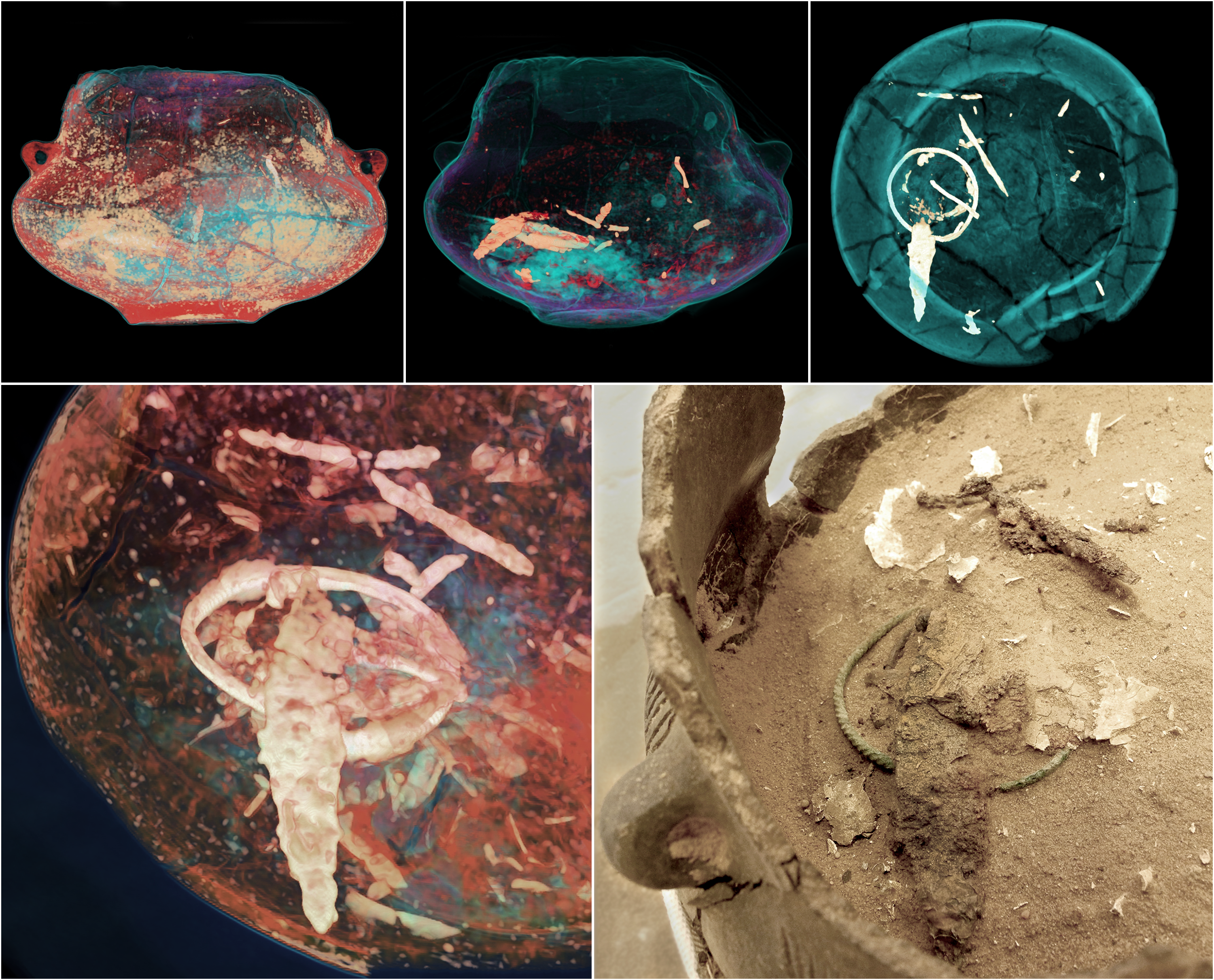

Supplement: S1 Fig — (TIF) [file pone.0274068.s002.tif]

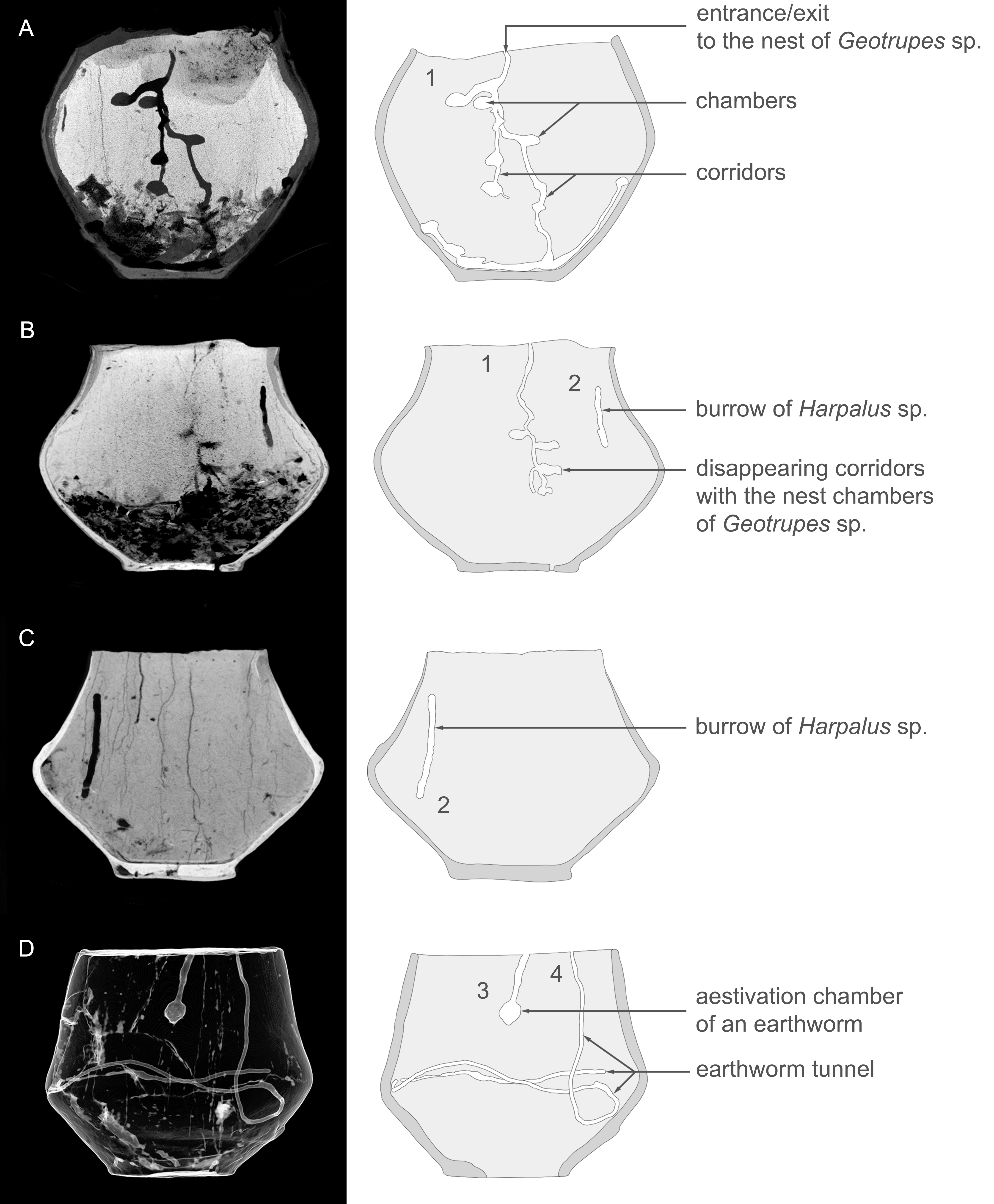

Supplement: S2 Fig — Visualisation of the urn and additional vessel fills (urn B from grave 632 (A), urn from grave 485 (B), urn from grave 599 (C), additional vessel from grave 600 (D)) with a preserved nests of the genus Geotrupes sp. (1), burrows of the genus Harpalus sp. (2), aestivation chamber (3) and tunnels of earthworms (4). (TIF) [file pone.0274068.s003.tif]
